# Glycofullerenes Inhibit Particulate Matter Induced Inflammation and Loss of Barrier Proteins in HaCaT Human Keratinocytes

**DOI:** 10.3390/biom10040514

**Published:** 2020-03-28

**Authors:** Chiang-Wen Lee, Yu-Han Su, Yao-Chang Chiang, I-Ta Lee, Sin-Yu Li, Hui-Chun Lee, Lee-Fen Hsu, Yi-Ling Yan, Hsing-Yen Li, Ming-Chun Chen, Kuo-Ti Peng, Chian-Hui Lai

**Affiliations:** 1 Department of Orthopaedic Surgery, Chang Gung Memorial Hospital, Puzi City, Chiayi County 61363, Taiwan; cwlee@gw.cgust.edu.tw (C.-W.L.); yaochang.chiang@gmail.com (Y.-C.C.); 0426cyndi@gmail.com (S.-Y.L.); 2 Department of Nursing, Division of Basic Medical Sciences, and Chronic Diseases and Health Promotion Research Center, and Research Center for Chinese Herbal Medicine, Chang Gung University of Science and Technology, Puzi City, Chiayi County 61363, Taiwan; hcli@cgust.edu.tw (H.-C.L.); lfhsu@mail.cgust.edu.tw (L.-F.H.); iling410@hotmail.com (Y.-L.Y.); 3 Department of Safety Health and Environmental Engineering, Ming Chi University of Technology, New Taipei City 24301, Taiwan; 4 College of Medicine, Chang Gung University, Guishan Dist., Taoyuan City 33303, Taiwan; 5 Graduate Institute of Biomedical Engineering, National Chung Hsing University, Taichung 40227, Taiwan; goat2658@gmail.com (Y.-H.S.); black910255@gmail.com (H.-Y.L.); daniel0044789@gmail.com (M.-C.C.); 6 School of Dentistry, College of Oral Medicine, Taipei Medical University, Taipei 11031, Taiwan; itlee0128@tmu.edu.tw; 7 Department of Respiratory Care, Chang Gung University of Science and Technology, Puzi City, Chiayi County 61363, Taiwan; 8 Division of Neurosurgery, Department of Surgery, Chang Gung Memorial Hospital, Puzi City, Chiayi County 61363, Taiwan; 9 Department of Medicinal and Applied Chemistry, Kaohsiung Medical University, Kaohsiung 80708, Taiwan

**Keywords:** glycofullerenes, particulate matter, reactive oxygen species, human keratinocytes, antioxidant, anti-inflammation

## Abstract

Exposure to particulate matter (PM) has been linked to pulmonary and cardiovascular dysfunctions, as well as skin diseases, etc. PM impairs the skin barrier functions and is also involved in the initiation or exacerbation of skin inflammation, which is linked to the activation of reactive oxygen species (ROS) pathways. Fullerene is a single C_60_ molecule which has been reported to act as a good radical scavenger. However, its poor water solubility limits its biological applications. The glyco-modification of fullerenes increases their water solubility and anti-bacterial and anti-virus functions. However, it is still unclear whether it affects their anti-inflammatory function against PM-induced skin diseases. Hence, glycofullerenes were synthesized to investigate their effects on PM-exposed HaCaT human keratinocytes. Our results showed that glycofullerenes could reduce the rate of PM-induced apoptosis and ROS production, as well as decrease the expression of downstream mitogen-activated protein kinase and Akt pathways. Moreover, PM-induced increases in inflammatory-related signals, such as cyclooxygenase-2, heme oxygenase-1, and prostaglandin E2, were also suppressed by glycofullerenes. Notably, our results suggested that PM-induced impairment of skin barrier proteins, such as filaggrin, involucrin, repetin, and loricrin, could be reduced by pre-treatment with glycofullerenes. The results of this study indicate that glycofullerenes could be potential candidates for treatments against PM-induced skin diseases and that they exert their protective effects via ROS scavenging, anti-inflammation, and maintenance of the expression of barrier proteins.

## 1. Introduction

Ambient air pollution (AAP), particularly with fine particulate matter (PM), has been reported to be harmful to human health by the World Health Organization (WHO) [1] and was responsible for 4.2 million deaths in 2016 [1]. AAP is greatly associated with various diseases, such as chronic obstructive pulmonary disease, cancer, cardiovascular diseases, and stroke [1]. Air pollutants can be produced by a variety of sources, including industries, households, fuel combustion by vehicles, or the burning of biomass. PM can be divided into categories based on the particle size, namely ultrafine particles (particle size of < 0.1 μm, UFP), fine particles (particle size of < 2.5 μm, PM_2.5_), and coarse particles (particle size of < 10 μm, PM_10_) [2]. The level of PM_2.5_ in the air on the weather forecast is an important daily environmental indication. The polyaromatic hydrocarbon coating on PM particles can cause inflammation by increasing oxidative stress levels and activating downstream mitogen-activated protein kinase (MAPK) signaling pathways. The skin constitutes the first line of defense of the human body against air pollutants, as the skin is the largest and outermost organ of the body. Notably, PM exposure has also been associated with skin aging, cancer, and inflammatory or allergic skin conditions, such as atopic dermatitis, eczema, psoriasis, or acne [3]. The increased levels of reactive oxygen species (ROS) and inflammatory factors, as well as a decreased expression of barrier proteins, are hypothesized to play a role in the pathological mechanisms of PM-induced skin diseases [4,5].

**Scheme 1:**
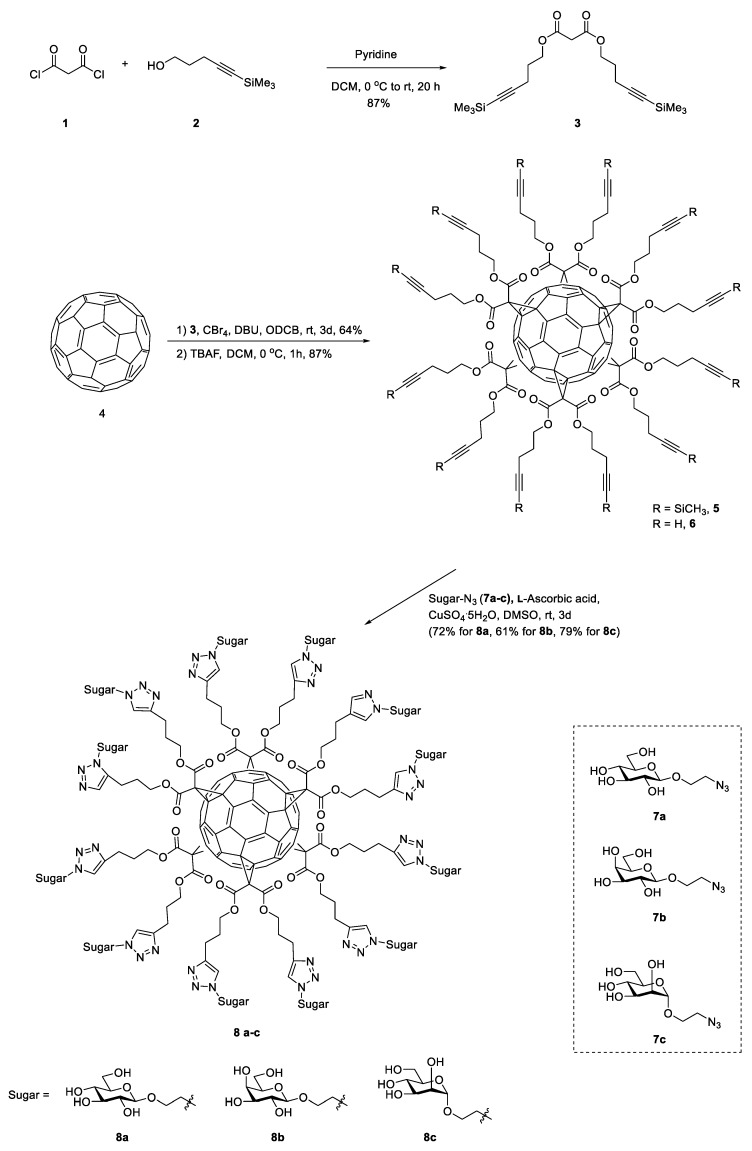
The synthesis procedure of the glycofullerenes **8a C_60_(Glc)_12_**, **8b C_60_(Gal)_12_**, and **8c C_60_(Man)_12_**.

Fullerene (C_60_) is a good radical scavenger, with a single C_60_ molecule able to interact and add up to 34 methyl radicals [6]. However, its poor water solubility limits its biological applications. Fullerene was discovered by Smalley and co-workers in 1985 [7], with the first macroscopic synthesis taking place in 1990 [8]. Moreover, there has been a rapid increase in attention regarding chemically functionalized fullerenes, due to their different applications [9,10]. Fullerenes entrapped in polyvinylpyrrolidone (PVP), resulting in a water-soluble compound which was termed a ‘radical sponge’ and was expected to act as an anti-ultraviolet A (UVA)-preventive agent in human skin keratinocytes [11]. Fullerene nanoparticles (NPs) have been used for dermatological and cosmetic applications (skin whitening, sunscreen, etc.) due to their high reactivity with ROS [12]. Notably, water-soluble fullerenes with direct functional derivatives have had a significant impact in the biological field [13,14] by exhibiting superior membranotropic functions, greater biological affinity to certain nucleic acids and proteins, as well as an increase in their ROS scavenging activity in target cells or tissues [14]. Water-soluble fullerenes were also shown to protect cell growth from various toxins, as well as promote recovery after ischemic stroke [10,15,16,17]. Of particular interest are fullerene derivatives substituted with sugar residues, known as glycofullerenes [17,18]. ROS damage is potentially prevented in the case of hexa-substituted glycol-fullerene derivatives, which have a globular structure and a T*_h_*-symmetrical octahedral addition pattern [18]. The synthesis of these hexa-substituted glycofullerenes could be achieved via a chemical reaction method, namely Cu(I) alkyne–azide cycloaddition (CuAAC), which is a very powerful synthetic tool for the functionalization of alkyne-fullerene building blocks **6** (as shown in Scheme 1) [18]. Dodecavalent galactoside fullerene-based glycoclusters has been synthesized and showed to have promising multivalent interactions with bacterial lectin PA-IL and a 12,000-fold binding ability [19]. Similarly, the dodecavalent heptoside fullerene sugar ball showed good inhibition of heptosyltransferase WaaC, a glycosyltransferase which catalyzes the incorporation of the first L-heptose into lipopolysaccharides [20]. Moreover, mannosylated fullerene sugar balls have been shown to have antiviral activity in an Ebola pseudotyped infection model [18,21,22]. A classical 3-(4,5-Dimethylthiazol-2-yl)-2,5- diphenyltetrazolium bromide (MTT) assay has also been recently performed using various fullerene sugar balls and the results indicated that they did not exert cytotoxicity [18]. 

The HaCaT cell line was first generated by Boukamp et. al. [23] and it can be a reliable differentiation model to estimate the inflammatory and repair response, as well as a cell platform for the development of novel treatments and the investigation of disease mechanisms [5,24,25,26,27,28]. In our previous study [27,29], we prepared water-soluble 7, 3′, 4′-trihydroxyisoflavone NPs by using the planetary ball mill method [27] and eupafolin NP delivery system [25,29] to investigate their individual antioxidant and anti-inflammatory activities in PM-induced skin inflammation [5,24,25,29]. The results of our previous study suggested that PM-induced skin cell inflammation regulated the AhR/p47 phox/NADPH oxidase pathway, and subsequently increased ERK1/2, p38/nuclear factor kappa-light-chain-enhancer of activated B cells (NF-κB), and c-Jun N-terminal kinase (JNK)/Activator protein 1 (AP-1) levels [5]. Moreover, it also upregulated the expression of cyclooxygenase-(COX-2) and prostaglandin E2 (PGE2) and downregulated filaggrin, thereby resulting in the impaired ability of barrier proteins, as well as skin function. ROS scavengers (*N*-acetylcysteine) and the apocynin (a Nox2 inhibitor) could suppress PM-induced ROS production. Fullerene has been shown to have radical scavenger activity. However, the biofunctions of glycofullerenes, especially their protective effects against PM-induced skin cell inflammation and expression of barrier protein have not yet been studied.

In order to further investigate this manner, we tried to develop and synthesize three different glycofullerenes, based on the CuAAC reaction, which differed based on their sugar substituent and had glucosides, galactosides, and mannosides substitutions, resulting in **8a C_60_(Glc)_12_**, **8b C_60_(Gal)_12_**, and **8c C_60_(Man)_12_** (Scheme 1). Moreover, we investigated their ability to attenuate PM-induced oxidative stress and inflammation in HaCaT keratinocytes. These glycofullerenes were characterized by nuclear magnetic resonance spectroscopy (NMR), Fourier-transform infrared spectroscopy (FTIR), ultraviolet–visible spectroscopy (UV–Vis), dynamic light scattering (DLS), and zeta-potential at each step of the synthesis process. In all of the biological experiments, the fullerenes which were not soluble in water (represented as **C_60_-H_2_O**) were used as a comparison. Annexin V-Fluorescein isothiocyanate (FITC)/propidium iodide staining was used to detect the stage of HaCaT cell apoptosis by flow cytometry. The typical molecular biological mechanism study models for antioxidant and anti-inflammation pathways were applied. Regarding the ability of the skin to repair, the expression of proteins which have been reported to be disrupted by PM [5], including loricrin, filaggrin, involucrin, and repetin, was also investigated. 

## 2. Materials and Methods 

### 2.1. Materials 

PM (Standard Reference Material 1649b, SRM-1649b) was purchased from the National Institute of Standards and Technology (Gaithersburg, MD, USA). Fullerene, tetrabutylammonium hydroxide (TBAH), sodium hydroxide (NaOH), malonyl dichloride, tetrabromomethane (CBr_4_), tetra-*n*-butylammonium fluoride (TBAF), 1,8-diazabicyclo [5.4.0]undec-7-ene (DBU), L-Ascorbic acid, CuSO_4_^.^5H_2_O, magnesium sulfate (MgSO_4_), and Sephadex LH-20 resin were purchased from Sigma-Aldrich (St. Louis, MO, USA). All solvents including toluene (Tol), dimethyl sulfoxide (DMSO), methanol (MeOH), pyridine (Py), 1,2-dichlorobenzene (ODCB), dichloromethane (DCM), tetrahydrofuran (THF), and methanol (MeOH) were dried and distilled by standard techniques. Analytical thin-layer chromatography (TLC) was performed on precoated plates (Silica Gel 60). Silica gel 60 (E. Merck, Darmstadt, Germany) was employed for all flash chromatography. All reactions were carried out in oven-dried glassware (100 °C) under an atmosphere of nitrogen unless indicated otherwise. Low-resolution and high-resolution mass spectra were recorded under Electrospray Ionization Time-of-Flight (ESI-TOF) mass spectra conditions. ^1^H and ^13^C NMR spectra were recorded on Varian Mercury-400 MHz. Chemical shifts are expressed in ppm using residual CDCl_3_ (7.24 ppm) or CD_3_OD (3.31 ppm) or D_2_O (4.67 ppm at 298 k) as internal standard. Fourier-transform infrared spectroscopy (FTIR) spectra were recorded on a Thermo Nicolet iS5 FTIR spectrometer (Thermo Fisher Scientfic Inc., Waltham, MA, USA). The absorption spectra of the fullerene derivatives were measured by Thermo Genesys 6 UV–Visible (Thermo Fisher Scientfic Inc., Waltham, MA, USA) spectrophotometer. HaCaT cells were purchased from AddexBio (San Diego, CA, USA). All buffers and solutions were prepared by using Millipore water. Dulbecco’s modified Eagle medium (DMEM) was obtained from GIBCO (Grand Island, NY, USA). Fetal bovine serum (FBS) was purchased from Hazelton Product (Denver, PA, USA). The bicinchoninic acid (BCA) protein assay kit was obtained from Pierce (Rockford, IL, USA). Western blotting enhanced chemiluminescence (ECL) detection kit and Hyperfilms were purchased from GE Healthcare Biosciences (Buckinghamshire, UK). Antibodies for Western blotting (total and phospho-p38, ERK, JNK, and phospho-Akt) were purchased from Cell Signaling Technology (Danvers, MA, USA). Total-Akt and cytosolic phospholipase A2 (cPLA2) antibodies were purchased from Santa Cruz Biotechnology (Dallas, TX, USA). Metalloproteinase-9 (MMP-9), involucrin, and repetin antibodies were purchased from Proteintech Group Inc. (Rosemont, IL, USA). Heme oxygenase-1 (HO-1) and COX-2 antibodies were obtained from Abcam (Cambridge, UK). Anti-loricrin antibody was purchased from Boster (Pleasanton, CA, USA). Filaggrin antibody was purchased from Genetex (Hsinchu, Taiwan). Anti-GAPDH antibody was purchased from Biogenesis (Boumemouth, UK). All of other chemicals were ACS reagent grade and purchased from Sigma-Aldrich (St. Louis, MO, USA). 

### 2.2. Synthesis of Bis(5-(Trimethylsilyl)pent-4-yn-1-yl) Malonate (**3**)

Malonyl dichloride **1** (0.50 mL, 5.14 mmol) was dropwisely added to a solution of 5-(trimethylsilyl)-4-pentyn-1-ol **2** (1.87 mL, 10.28 mmol), pyridine (0.84 mL, 10.33 mmol), and dry DCM (73 mL) at 0 °C for stirring 1 h. The reaction mixture was then warmed to room temperature and stirred for 19 h. The solution was evaporated and co-evaporated with DCM three times. The crude was purified by column chromatography to afford **3** (1.70 g, 87%). ^1^H NMR (400 MHz, CDCl_3_) δ 4.22 (t, *J* = 6.3 Hz, 4H), 3.36 (s, 2H), 2.30 (t, *J* = 7.0 Hz, 4H), 1.85 (p, *J* = 6.7 Hz, 4H), 0.12 (s, 18H). NMR spectrum of **3** is also provided in Figure S1 in Supplementary Data.

### 2.3. Synthesis of Dodecatrimethylsilylalkyne-Fullerene (**5**)

To a solution of fullerene **4** (201.8 mg, 0.28 mmol) in *O*-dichlorobenzene (ODCB) (6.0 mL) was added **3** (1.07 g, 2.80 mmol), CBr_4_ (9.29 g, 28.0 mmol), and 1,8-diazabicyclo[5.4.0]undec-7-ene (DBU) (0.84 mL, 5.60 mmol). The reaction mixture was stirred at room temperature for 3 days. The solvent was evaporated in vacuoi, then the crude was purified by column chromatography to afford **5** (527.9 mg, 64%). IR (neat): 2177 (C≡C), 1747 (C=O); ^1^H NMR (400 MHz, CDCl_3_) δ 4.62–4.17 (m, 24H), 2.53–2.15 (m, 24H), 2.09–1.74 (m, 24H), 0.12 (s, 108H). NMR, IR, and UV absorption spectrum of **5** were also provided in Figures S2–S4 in Supplementary Data.

### 2.4. Synthesis of Dodecaalkyne-Fullerene (**6**)

A 1M solution of TBAF in THF (2.1 mL, 7.50 μmol) was dropwisely added to a solution of **5** (448.8 mg, 0.15 mmol) in dry DCM (15 mL) at 0 °C for stirring 1 h. The solution was diluted with DCM, washed with water, dried over MgSO_4_, and concentrated under reduced pressure. The crude was purified by column chromatography to afford **6** (276.4 mg, 87%). IR (neat): 3288 (≡C-H), 2116 (C≡C), 1742 (C=O); ^1^H NMR (400 MHz, CDCl_3_) δ 4.61–4.21 (m, 24H), 2.43–2.19 (m, 24H), 2.07–1.76 (m, 36H). NMR, IR, and UV absorption spectrum of **6** were also provided in Figures S5–S7 in Supplementary Data.

### 2.5. Synthesis of Glycofullerenes: 8a C_60_(Glc)_12_, 8b C_60_(Gal)_12,_ 8c C_60_(Man)_12_

Compound **8a C_60_(Glc)_12_:** To a reaction mixture of **6** (38.0 mg, 17.87 μmol) and **7a** (97.2 mg, 0.39 mmol) in DMSO (7 mL) was added L-ascorbic acid (7.9 mg, 44.68 μmol) and CuSO_4_^.^5H_2_O (3.6 mg, 14.30 μmol) and stirred at room temperature for 3 d. The reaction mixture was concentrated in vacuoi. The crude was purified by Sephadex LH-20 to afford **8a** (65.8 mg, 72%). IR (neat): 3375 (O-H), 1736 (C=O); ^1^H NMR (400 MHz, DMSO) δ7.92 (s, 12H), 5.06 (s, 10H), 4.98–4.88 (m, 17H), 4.59–4.15 (m, 77H), 4.05 (s, 14H), 3.86 (s, 12H), 3.65 (s, 11H), 3.49–3.38 (m, 14H), 3.20–2.90 (m, 41H), 2.71 (d, *J* = 37.5 Hz, 31H), 2.21 (s, 29H), 2.06–1.65 (m, 48H); HRMS (ESI): calcd for C_234_H_245_N_36_O_96_Na [M + Na]^+^: 5117.5293; found: 5140.4565. Synthetic route of 7a were showed in Figure S8. NMR, IR, UV absorption and mass spectrum of **8a** were also provided in Figures S9–S12 in Supplementary Data.

Compound **8b C_60_(Gal)_12_**: Similar experiment procedure as described for **8a**. The reaction contained with **6** (40.0 mg, 18.81 μmol), **7b** (102.2 mg, 0.41 mmol), L-ascorbic acid (8.3 mg, 47.03 μmol) and CuSO_4_^.^5H_2_O (3.8 mg, 15.05 μmol) in DMSO (8 mL) at room temperature for 3 d. The reaction mixture was concentrated in vacuoi. The crude was purified by Sephadex LH-20 to afford **8b** (58.7 mg, 61%). IR (neat): 3383 (O-H), 1738 (C=O); ^1^H NMR (400 MHz, DMSO) δ 7.90 (s, 12H), 4.93 (s, 12H), 4.72 (s, 12H), 4.66–4.42 (m, 36H), 4.37 (s, 24H), 4.20–4.11 (m, 12H), 4.04 (s, 12H), 3.83 (s, 12H), 3.61 (s, 12H), 3.57–3.40 (m, 24H), 3.28 (s, 24H), 2.66 (s, 24H), 1.96 (s, 24H); HRMS (ESI): calcd for C_234_H_245_N_36_O_96_Na [M + Na]^+^: 5117.5294; found: 5117.5535. Synthetic route of 7b were showed in Figure S8R, IR, UV absorption and mass spectrum of **8b** were also provided in Figures S13–S16 in Supplementary Data.

Compound **8c C_60_(Man)_12_**: Similar experiment procedure as described for **8a**. The reaction contained with **6** (37.4 mg, 18.81 μmol), **7c** (97.2 mg, 0.39 mmol), L-Ascorbic acid (7.7 mg, 43.98 μmol) and CuSO_4_^.^5H_2_O (3.5 mg, 14.07 μmol) in DMSO (7 mL) at room temperature for 3 d. The reaction mixture was concentrated in vacuoi. The crude was purified by Sephadex LH-20 to afford **8c** (71.1 mg, 79%). IR (neat): 3397 (O-H), 1735 (C=O); ^1^H NMR (400 MHz, DMSO) δ7.90 (s, 12H), 4.71 (m, 24H), 4.55 (m, 29H), 4.44 (m, 40H), 4.28 (m, 15H), 3.87 (s, 12H), 3.72 (s, 12H), 3.51 (m, 48H), 3.05 (m, 12H), 2.51 (m, 24H), 1.91 (m, 24H); HRMS (ESI): calcd for C_234_H_246_N_36_O_96_Na [M + Na]^+^: 5118.5372; found: 5118.6387. Synthetic route of 7c were showed in Figure S8. NMR, IR, UV absorption and mass spectrum of **8c** were also provided in Figures S17–S20 in Supplementary Data. 

### 2.6. Transmission Electron Microscopy Analysis of Glycofullerene Particle Size 

Transmission electron microscopy (TEM) was performed by using a JEOL microscope (Model JEM-2100) (JEOL Ltd., Tokyo, Japan) operated at 200 keV to analyze the sizes and dispersion of the synthetic glycofullerenes. A drop of the glycofullerenes solution (~1 μL) was dropped on a carbon-coated 200-mesh copper grid. The grid was left to dry at room temperature for hours. Before the TEM analysis, the grid was then further dried under vacuum overnight.

### 2.7. Particle Size of Glycofullerene Analysis by Dynamic Light Scattering and Zeta-Potential Measurement

The hydro-diameter size and zeta potential of the glycofullerene particles were analyzed using a particle analyzer with dynamic light scattering (DLS) technique (Horiba, SZ-100V2, Kyoto, Japan) at 25 °C with a laser angle of 90°. The samples were diluted with pure water. Each value was measured in triplicate.

### 2.8. Cell Culture Condition

Human epidermal keratinocyte (HaCaT) cells were purchased from AddexBio (San Diego, CA, USA). The HaCaT cell line was seeded in a 12 well plate (2 × 10^5^ per well) with 1 mL Dulbecco’s Modified Eagle Medium (DMEM, Gibico, Grand Island, NY, USA) supplemented with 10% FBS (Hazelton Research Products, Denver, PA, USA) and 1% penicillin–streptomycin (Gibico, USA), and incubated at 37 °C in a humidified atmosphere containing 5% CO_2_/95% air. After cells were seeded for 24 h to ensure that they were stable, the medium was renewed and followed experiments were performed. Cells were treated with 0.05% (*w*/*v*) trypsin/0.53 mM EDTA for 5 min at 37 °C, when cultures reached confluence. The cell viability was also simultaneously tested using the 3-(4,5-Dimethylthiazol-2-yl)-2,5-diphenyltetrazolium bromide (MTT) assay, to ensure a survival rate of at least 90% with each passage. The HaCaT cells passages within 5 to 12 were used for preforming experiments.

### 2.9. Measurement of Cell Apoptosis 

The flow cytometry with the Annexin V-fluorescein isothiocyanate (FITC)/propidium iodide (Thermo Fisher Scientific, Waltham, MA, USA) staining were used for measuring the cell apoptosis. Different types of glycofullerenes (1 μM) were dissolved with pure water and pre-treated to HaCaT cells for 1 h, then treated with PM (SRM-1649b) with 50 μg/cm^2^ for another 24 h. After 24 h of PM administration, these of cells were followed to stain with the dye—Annexin V-FITC and propidium iodide (for 30 min), according to the manual, and subjected to detect by flow cytometry (Accuri C6, BD Biosciences, San Jose, CA, USA). Data were repeated and collected from at least three independent experiments.

### 2.10. Determination of ROS Productions from Cellular and Mitochondrial Areas

The ROS production from cellular area was measured using the 2′,7′-dichlorodihydrofluorescein diacetate (H2DCFDA) and CellROX assay. HaCaT cells were seeded onto 12-well plates (2 × 10^5^ per well) and incubated 24 h for stable. Cells were treated with different types of glycofullerene (1 μM) for 1 h and then administered PM (50 μg/cm^2^) for another 2 h. Cells were stained with H2DCFDA and CellROX reagents (Thermo Fisher Scientific, USA). The fluorescence intensity of the cells was measured using flow cytometry (excitation/mission wavelength, 488/530 nm). Furthermore, MitoSOX^TM^ (Molecular Probes, Eugene, OR, USA) was used for assaying the levels of the mitochondrial ROSs with the same treatment schedule as mentioned above. The excitation wavelength and emission wavelength for flow cytometry were set as 488 and 585 nm, respectively. The data were collected from at least three times of independent experiments. 

### 2.11. Immunoblotting for MAPKs and Epidermal-Related Proteins Measurement

HaCaT after being seeded and incubated onto 12-well plates (2 × 10^5^ per well) for 24 h, were pre-treated with 1 μM of different types of glycofullerenes (1 μM) for 1 h and then exposed to PM (50 μg/cm^2^) for another 6 h (kinases) or 24 h (inflammatory- and protection-related proteins). After treatment, the cells were lysed with lysis buffer for protein extraction. Equal amounts of the protein were loaded and separated by electrophoresis in the SDS-polyacrylamide gel (10–12.5% polyacrylamide) and then transferred onto nitrocellulose membranes. The primary antibodies, phospho-p38, ERK, JNK and phospho-Akt, cPLA2, MMP-9, involucrin and repetin, HO-1 and COX-2, loricrin, and filaggrin were utilized to measure the expression levels of these of targets. Next, the horseradish peroxidase conjugated secondary antibodies with were added, and the enhanced chemiluminescence (ECL) detection system was used to assess the immunoreactivities. The signals were detected on a ChemiDoc^TM^ XRS+ image system (Bio-Rad Laboratories, Hercules, CA, USA) and GAPDH signal was used as a loading control. At least three independent replicates with a similar pattern of blotting data were collected. The values were calculated from those of figures, then a figure with closer pattern of statistical values was selected for presentation.

### 2.12. Measurement of Prostaglandin E2 Production

HaCaT cells were cultured in 12-well culture plates (2 × 10^5^ per well) and incubated for 24 h to ensure stale for followed experiments. After reaching confluence, cells were pre-treated with 1 μM of different types of glycofullerene for 1 h and then exposed to PM (50 μg/cm^2^) for another 24 h. After treatment, medium was collected and stored at –80 °C until assays were performed. PGE2 levels were measured by PGE2 enzyme immunoassay kit (Cayman Chemical, Ann Arbor, MI, USA), according to the manufacturer’s instructions. Experiments were repeated at least three times and the data were collected.

### 2.13. Statistical Analysis

Data were expressed as mean ± standard error of the mean (SEM). One-way ANOVA followed by post-hoc Tukey’s multiple comparison (multiple groups) were used to analyze the data with version 5 of GraphPad Prism software (GraphPad, San Diego, CA, USA). A *p*-value < 0.05 was considered significant.

## 3. Results

### 3.1. Synthesis and Characterization of Glycofullerenes

**Figure 1:**
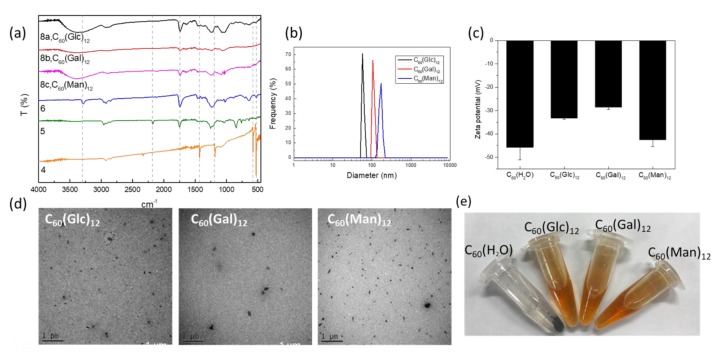
(**a**) FTIR, (**b**) dynamic light scattering (DLS), (**c**) zeta-potential, and (**d**) TEM data for **C_60_(Glc)_12_**, **C_60_(Gal)_12_**, and **C_60_(Man)_12_**, respectively. (**e**) Images of the newly synthesized glycofullerenes dissolved in water. **C_60_-H_2_O** formed a precipitate in the Eppendorf tube.

The steps required for the synthesis of the glycofullerenes **8a C_60_(Glc)_12_**, **8b C_60_(Gal)_12_**, and **8c C_60_(Man)_12_** are shown in Scheme 1 (synthetic route, UV, NMR, and Mass spectra are shown in the Supplementary Data, Figures S1–S20). The reaction of malonyl dichloride **1** with 5-(trimethylsilyl)-4-pentyn-1-ol **2** in the presence of pyridine led to the production of malonate **3** with a yield of 87 %. The hexakis-adduct (dodecatrimethylsilylalkyne-fullerene) **5** was then synthesized via the reaction between fullerene C_60_ **4** (1 equiv.) and malonate **3** (10 equiv.), CBr_4_ (100 equiv.), and DBU (20 equiv.) in *o*-dichlorobenzene (ODCB) at room temperature for 3 days. The resulting product was then purified on a fresh silica-gel column to obtain **5**, with a 64% yield. Subsequently, treatment of **5** with tetra-n-butylammonium fluoride (TBAF) in CH_2_Cl_2_ led to the synthesis of dodecaalkyne-fullerene **6** with an 87% yield. The ^1^H NMR spectrum of **6** indicates the disappearance of the trimethylsilyl (TMS)-protecting groups at ~0.12 ppm (Figure S5). Moreover, the chemical structure of the fullerenes derivatives was easily confirmed by FTIR analysis. As shown in Figure 1a, the fullerene C_60_ **4** powder in KBr shows a typical IR signal at ~1482, ~1180, ~575, and ~526 cm^−1^. The dodecatrimethylsilylalkyne-fullerene **5** has a signal at 2177 and 1747 cm^−1^ for the alkyne (C≡C) and carbonyl (C=O) groups, respectively. After removal of the TMS protecting group, compound **6** exhibits a terminal alkyne (≡C-H) C-H bond stretching signal at 3288 cm^−1^. 

To synthesize the glycofullerenes **8a C_60_(Glc)_12_**, **8b C_60_(Gal)_12_**, and **8c C_60_(Man)_12_** (Scheme 1), we used an optimized CuAAC method, known as the click chemistry strategy. Different glycofullerenes can be prepared relatively easy using this methodology, by reacting the twelve terminal alkynes on **6** with azido-sugar such as **7a** (Glc-N_3_), **7b** (Gal-N_3_), and **7c** (Man-N_3_) [18]. As such, the mixture of **6** (1 equiv.), **7** (**7a**, **7b,** or **7c**, 22 equiv.), CuSO_4_·5H_2_O (0.8 equiv.), and sodium ascorbate (2.5 equiv.) in DMSO was stirred at room temperature for 3 days. The crude products were purified by using a size exclusion Sephadex LH-20 column to obtain **8a C_60_(Glc)_12_**, **8b C_60_(Gal)_12_**, or **8c C_60_(Man)_12_**, in a yield of 72%, 61% or 79%, respectively. These glycofullerenes display 12 carbohydrate epitopes on their periphery, in a globular topology. The molecular weight of **8a C_60_(Glc)_12_**, **8b C_60_(Gal)_12_**, and **8c C_60_(Man)_12_** was recorded by using electrospray ionization (ESI) high-resolution mass spectrometry (Figures S12, S16, and S20). The FTIR spectrum (Figure 1a) indicated a broad single signal at around 3350 cm^−1^, a typical O-H stretching for the sugar moieties of **8a C_60_(Glc)_12_**, **8b C_60_(Gal)_12_**, and **8c C_60_(Man)_12_**, respectively.

The **8a C_60_(Glc)_12_**, **8b C_60_(Gal)_12_**, and **8c C_60_(Man)_12_** glycofullerenes were characterized regarding their hydrodynamic diameter (Figure 1b), zeta potential (Figure 1c) for their surface charge by DLS, and particle morphology by TEM (Figure 1d). The UV absorption spectra show almost identical bands between 190 and 290 nm for the fullerene derivatives **5**, **6**, **8a C_60_(Glc)_12_**, **8b C_60_(Gal)_12_**, and **8c C_60_(Man)_12_** (Figure S4–S19 in the Supplementary Data). The hydrodynamic diameters of **8a C_60_(Glc)_12_**, **8b C_60_(Gal)_12_**, and **8c C_60_(Man)_12_** were 69.3 ± 5.2, 103.2 ± 6.1, and 172.0 ± 17.7 nm, respectively. The zeta potential of **8a C_60_(Glc)_12_**, **8b C_60_(Gal)_12_**, and **8c C_60_(Man)_12_** in water were −33.23 ± 0.5, −28.57 ± 0.9, and −42.43 ± 3.0, respectively. As shown in Figure 1e, **8a C_60_(Glc)_12_**, **8b C_60_(Gal)_12_**, and **8c C_60_(Man)_12_** can be dissolved in an aqueous solution. In contrast, **C_60_-H_2_O** was not soluble in water and formed a precipitate in the Eppendorf tube.

### 3.2. Glycofullerenes Reduced the Ratio of PM-Induced Cell Apoptosis

**Figure 2:**
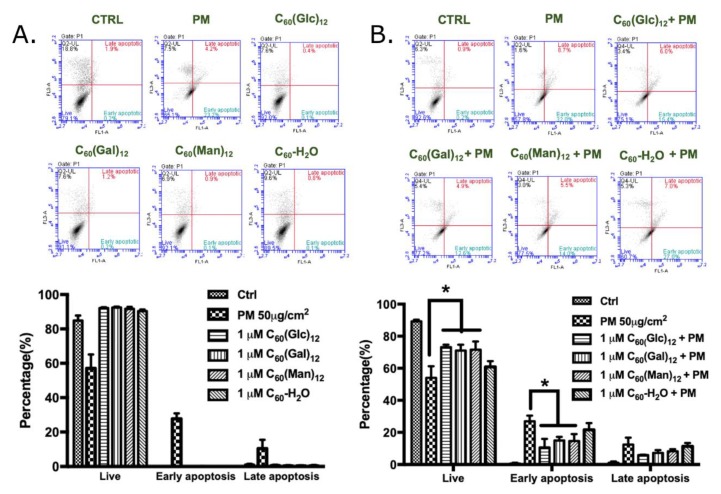
The effects of glycofullerenes and particulate matter (PM) on cell apoptosis in human skin keratinocyte (HaCaT) cells. (**A**) Glycofullerenes (1 μM) or PM (SRM 1649b, 50 μg/cm^2^) treatment of HaCaT cells for 24 h, (**B**) pre-treatment with glycofullerenes for 1 h and then incubation with PM for another 24 h. The images were obtained by flow cytometry using the Annexin V-FITC and propidium iodide staining. The upper panel of (**A**,**B**) show the raw data obtained by flow cytometry and the right lower quadrant of each raw figure indicates the level of early apoptosis in the cells. The bar graphs illustrate the cumulative counts obtained by flow cytometry. Data are expressed as mean ± standard error of mean (SEM) from at least three individual experiments. * *p* < 0.05 as compared to the PM exposure group.

Flow cytometry with Annexin V-FITC and propidium iodide staining at 24 h was used to determine cell apoptosis. The times for the apoptosis, ROS, and Western blot assays were based on our previous study on HaCaT cells [5]. Based on the properties of Annexin V-FITC and propidium iodide, this staining could be used to identify the early or late stages of apoptosis and necrosis by analyzing the combination of positive/negative signals of Annexin V-FITC and propidium iodide, a method which has been used to detect the different stages of apoptosis in HaCaT cells via flow cytometry [30,31,32]. As shown in Figure 2A, incubation with glycofullerenes and **C_60_-H_2_O** did not significantly induce HaCaT apoptosis, while PM did. Moreover, as observed in Figure 2B, pre-treatment for 1 h with 1 μM of **8a C_60_ (Glc)_12_**, **8b C_60_(Gal)_12_**, or **8c C_60_(Man)_12_** could reduce the ratio of early-stage of PM (SRM 1649b, 50 μg/cm^2^)-induced cell apoptosis, while the non-soluble fullerene **C_60_-H_2_O** did not show a notable inhibition effect on PM-induced apoptosis in HaCaT cells.

### 3.3. Glycofullerenes Suppressed PM-Induced Production of Cellular and Mitochondrial Reactive Oxygen Species 

**Figure 3:**
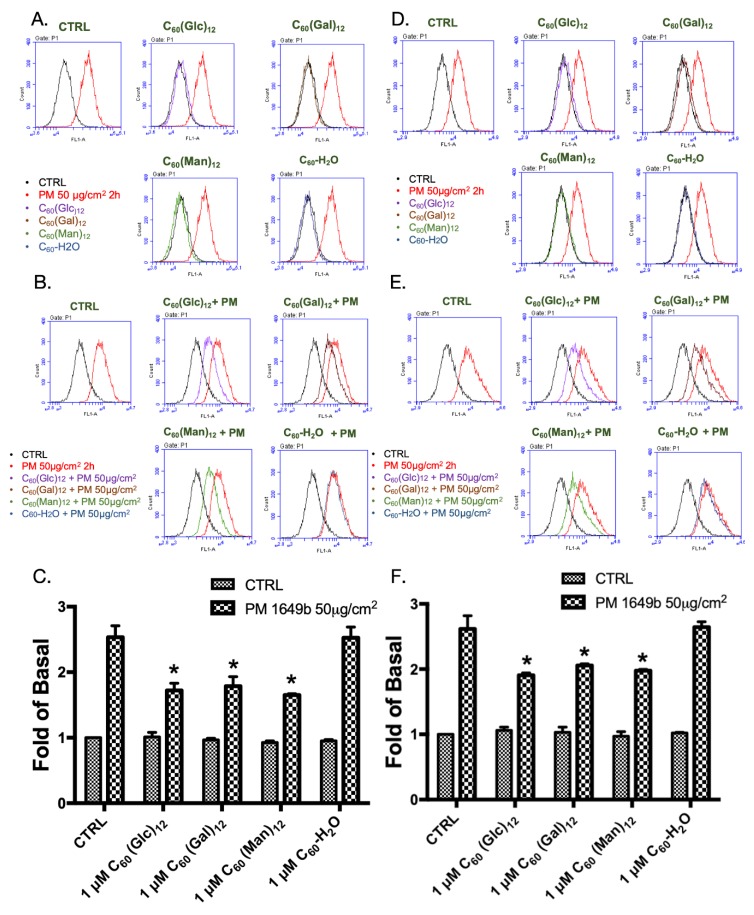
The cellular production of reactive oxygen species (ROS) following treatment with glycofullerenes and particulate matter (PM) in HaCaT cells. (**A**) graphs of flow cytometry raw data following treatment with glycofullerenes (1 μM) or PM (SRM 1649b, 50 μg/cm^2^) alone and (**B**) pre-treatment with glycofullerenes for 1 h followed by incubation with PM for another 2 h. The signals were detected by H2DCFDA staining. (**C**) The bar graphs illustrate the cumulative counts of H2DCFDA results obtained by flow cytometry. (**D**) Flow cytometry data from cells treated with only glycofullerenes or PM and (**E**) pre-treated with glycofullerenes for 1 h followed by incubation with PM for another 2 h. The signals were detected by using CellROX staining. (**F**) The bar graphs illustrate the cumulative counts of CellROX results obtained by flow cytometry. All bar graphs data were collected from at least three individual experiments and expressed as mean ± SEM. * *p* < 0.05 when compared to the PM exposure group.

**Figure 4:**
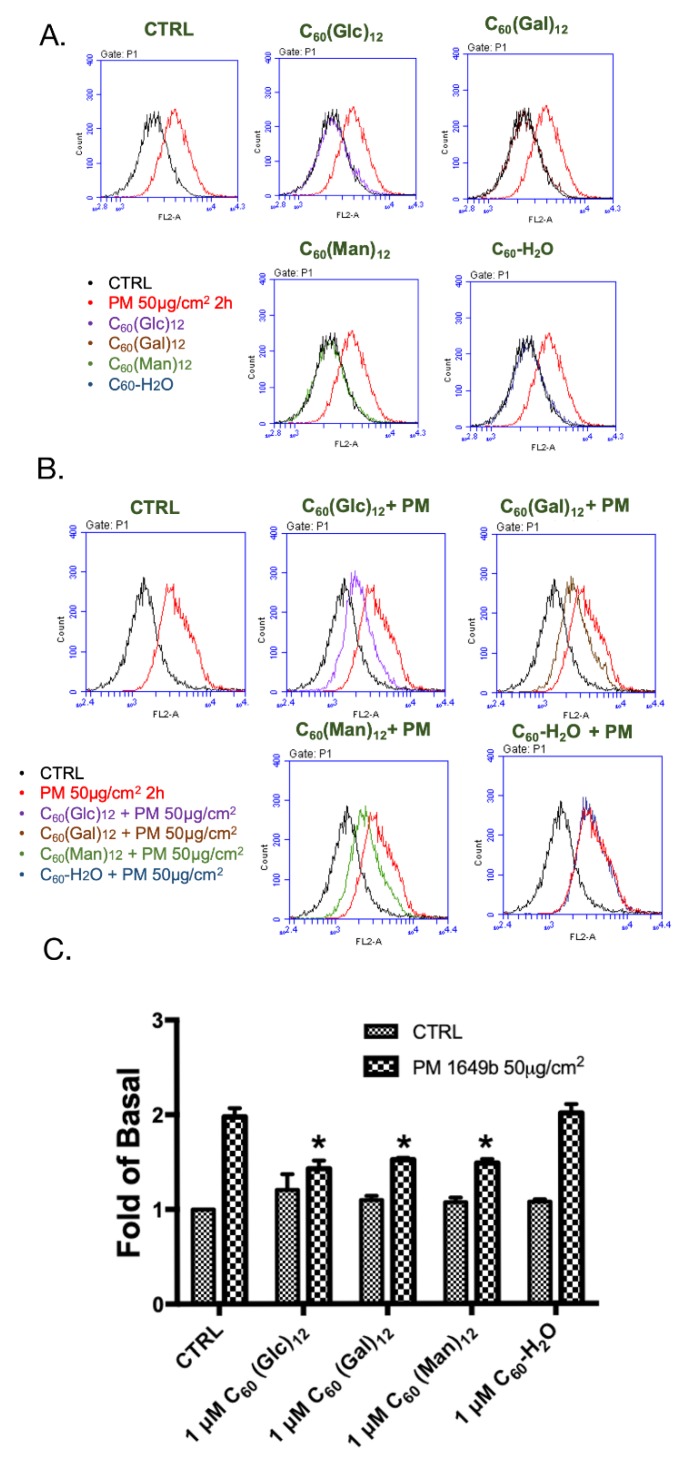
Effect of glycofullerenes and particulate matter (PM) on mitochondrial reactive oxygen species (ROS) production (determined by flow cytometry after staining with MitoSOX) in HaCaT cells. (**A**) Graphs of the raw flow cytometry data from cells treated with glycofullerenes (1 μM) or PM (SRM 1649b, 50 μg/cm^2^) alone and (**B**) pre-treated with glycofullerenes for 1 h followed by incubation with PM for another 2 h. (**C**) The bar graph illustrates the cumulative counts of MitoSOX results obtained by flow cytometry. All bar graphs data were collected from at least three individual experiments and expressed as mean ± SEM. * *p* < 0.05 when compared to the PM exposure group.

It is well known that PM could cause ROS accumulation and subsequently initiate the development of diseases [5,33]. Previously, fullerenes have been shown to have ROS scavenging activity, which could be enhanced by their water-soluble properties [14]. Hence, the effects of glycofullerenes on ROS generation in the cell and mitochondria were investigated. H2DCFDA and the CellROX Red dye were mainly used to measure the ROS production in the cytoplasm area by detecting the activity of hydroxyl and peroxyl groups (H_2_O_2_) (by H2DCFDA) and the oxidation of various ROS (by CellROX). MitoSox was used to detect the ROS in the mitochondria due to it is specific sensitivity to the superoxide produced by the mitochondria. As shown in Figure 3A, H2DCFDA, an agent used to determine cellular ROS, was used to stain the cells for flow cytometry, which were treated with 1 μM of **8a C_60_ (Glc)_12_**, **8b C_60_(Gal)_12_**, or **8c C_60_(Man)_12_**, and the non-soluble fullerene, **C_60_-H_2_O**. Our results showed that the cells treated with the indicated fullerenes had lower ROS production when compared with the vehicle (water) treated control group, while PM exposure significantly increased ROS levels. As expected, glycofullerenes were found to reduce PM-induced ROS production but the non-soluble fullerene, **C_60_-H_2_O** did not (Figure 3B). The bar graphs in Figure 3C illustrate the cumulative counts of the H2DCFDA flow cytometry results. A similar pattern was obtained by using another cellular ROS staining reagent—CellROX (Figure 3D–F). In addition, mitochondrial ROS levels after treatment with glycofullerenes and PM were detected by using flow cytometry with MitoSOX staining (Figure 4). As shown in Figure 4C, ROS levels were increased by PM treatment, while treatment with glycofullerenes alone did not induce mitochondrial ROS production. Moreover, pre-treatment with 1 μM of **8a C_60_(Glc)_12_**, **8b C_60_(Gal)_12_**, or **8c C_60_(Man)_12_** was shown to suppress PM-triggered mitochondrial ROS production in HaCaT cells. The results indicate that the PM-induced increase in cellular and mitochondrial ROS (H_2_O_2_ and superoxide) could be suppressed by pre-treatment with the **8a C_60_ (Glc)_12_**, **8b C_60_(Gal)_12_**, or **8c C_60_(Man)_12_** glycofullerenes, but not by the non-soluble fullerene, **C_60_-H_2_O**.

### 3.4. Glycofullerenes Suppressed the PM-Induced Phosphorylation of Mitogen-Activated Protein Kinases and Akt Proteins

**Figure 5:**
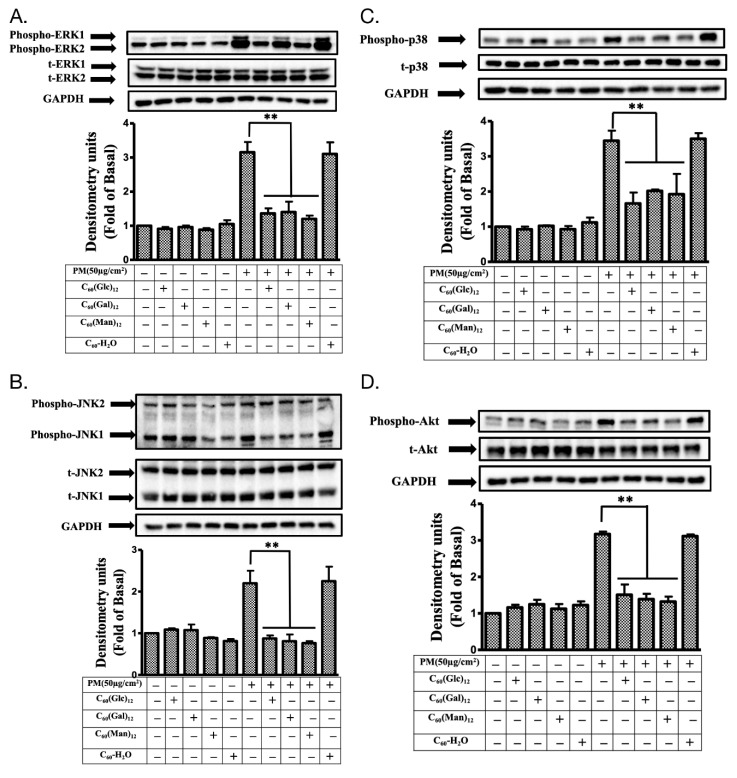
The phosphorylation of mitogen-activated protein kinase (MAPK) and Akt proteins in HaCaT cells treated with glycofullerenes and particulate matter (PM). The effects of the treatment on different kinases (**A**) extracellular signal-regulated kinase (ERK, pp42/pp44), (**B**) c-Jun N-terminal kinase (JNK), (**C**) p38, and (**D**) Akt were measured by immunoblotting in HaCaT cells with or without pre-treatment with 1 μM of **8a C_60_(Glc)_12_**, **8b C_60_(Gal)_12_**, **8c C_60_(Man)_12_**, or non-soluble fullerene, **C_60_-H_2_O**, respectively, for 1 h and then incubation with PM (SRM 1649b, 50 μg/cm^2^) for 6 h. Glyceraldehyde 3-phosphate dehydrogenase (GAPDH) was used as a loading control. The bar graphs were calculated from the phospho-ERK, JNK, p38, and Akt blot signals, respectively. The blots were representative of three independent experiments. The data were collected from at least three individual experiments and expressed as mean ± SEM. ** *p* < 0.01 when compared to the PM exposure group.

The mitogen-activated protein kinase (MAPK) and Akt (also known as protein kinase B) pathways play roles in various cellular processes and have been demonstrated to be possibly regulated by ROS [5,34]. As such, the effects of the glycofullerenes on the change in the protein levels of the MAPK and Akt pathways following PM exposure in a HaCaT cell model were further evaluated by immunoblotting methods. As shown in Figure 5, treatment with **8a C_60_(Glc)_12_**, **8b C_60_(Gal)_12_**, or **8c C_60_(Man)_12_** and the non-soluble fullerene, **C_60_-H_2_O** alone did not significantly alter the total and phosphorylated protein levels of extracellular signal-regulated kinase (ERK1/2, pp42, and pp44), P38, c-Jun N-terminal kinase 1/2 (JNK 1/2), and Akt. Moreover, the increased expression of phosphorylated ERK, P38, JNK, and Akt induced by PM exposure in HaCaT cells was significantly suppressed by pre-treatment with 1 μM of glycofullerenes, while pre-treatment with the non-soluble fullerene, **C_60_-H_2_O** alone did not influence those of phosphorylated protein levels.

### 3.5. Glycofullerenes Suppressed PM-Induced Changes in the Expression of Inflammatory Proteins

**Figure 6:**
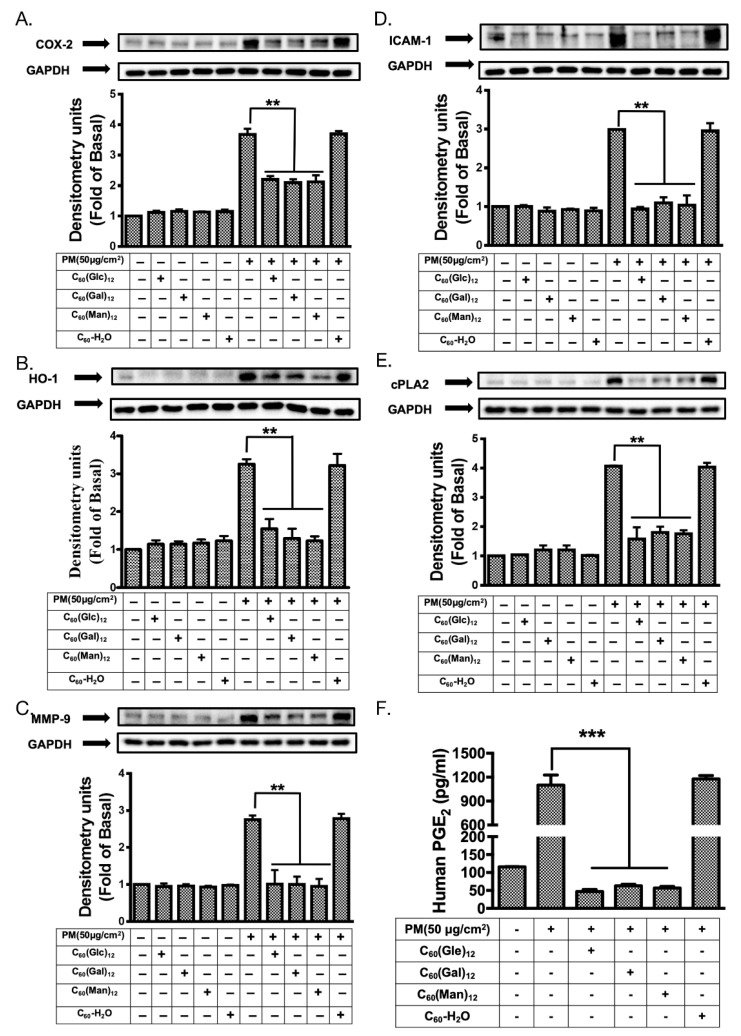
The effects of glycofullerenes and particulate matter (PM) treatment on inflammatory pathways-related proteins in HaCaT cells. Effects of the treatment on inflammatory pathways-related proteins (**A**) cyclooxygenase-2 (COX-2), (**B**) intercellular adhesion molecular-1 (ICAM-1), (**C**) heme oxygenase-1 (HO-1), (**D**) cytosolic phospholipase A2 (cPLA2), and (**E**) metalloproteinase-9 (MMP-9) were measured by immunoblotting and (**F**) prostaglandin E2 (PGE2) levels were measured by using an ELISA kit in HaCaT cells with or without pre-treatment with 1 μM of **8a C_60_(Glc)_12_**, **8b C_60_(Gal)_12_**, **8c C_60_(Man)_12_**, or non-soluble fullerene, **C_60_-H_2_O** for 1 h and then incubation with PM (SRM 1649b, 50 μg/cm^2^) for 24 h. Glyceraldehyde 3-phosphate dehydrogenase (GAPDH) was used as a loading control. The blots were representative of three independent experiments. The data were collected from at least three individual experiments and expressed as mean ± SEM. ** *p* < 0.01, *** *p* < 0.001 when compared to the PM exposure group.

ROS also trigger the expression of several inflammatory proteins and cause cell and tissue injury by regulating the cellular kinase pathways [5,35]. Therefore, the level of COX-2, intercellular adhesion molecular-1 (ICAM-1), HO-1, cPLA2, MMP-9, and PGE2 were measured after PM or glycofullerenes treatment. As shown in Figure 6, treatment with 1 μM of **8a C_60_(Glc)_12_**, **8b C_60_(Gal)_12_**, **8c C_60_(Man)_12_** or non-soluble fullerene, **C_60_-H_2_O** did not change the base level of COX-2, ICAM-1, HO-1, cPLA2, MMP-9, and PGE2 when compared with the vehicle-treated control. Notably, all the glycofullerenes were shown to significantly reduce PM-induced increases in COX-2, ICAM-1, HO-1, cPLA2, and MMP-9 expression, as well as PGE2 production. However, pre-treatment with non-soluble fullerene, **C_60_-H_2_O** could not decrease the PM-induced increase in the expression of inflammatory proteins.

### 3.6. Pre-Treatment with Glycofullerenes Maintained the Level of Protection Proteins in Keratinocytes under PM-Exposure

**Figure 7:**
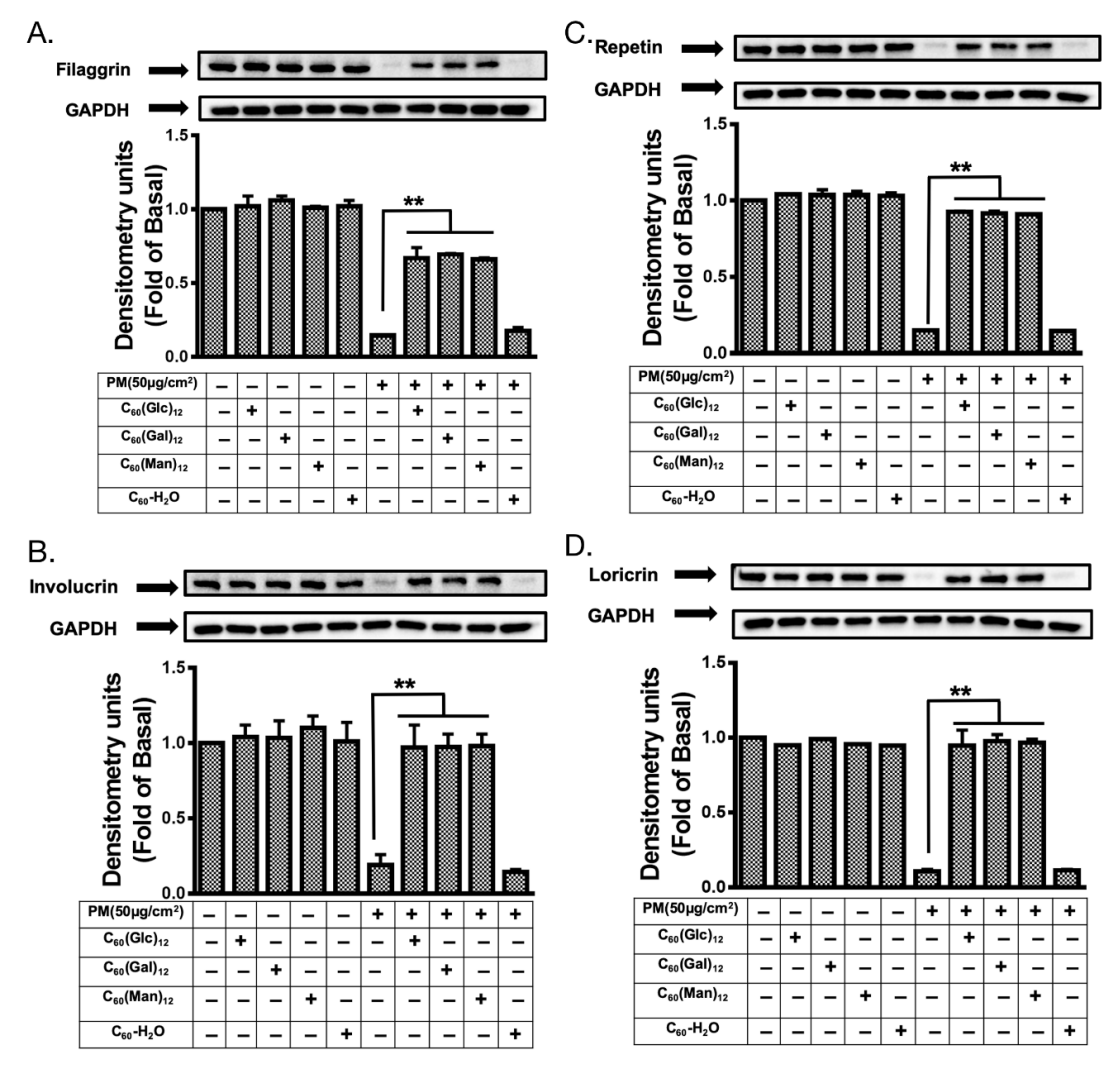
The expression levels of skin protection proteins in HaCaT cells treated with glycofullerenes and particulate matter (PM). The changes in the level of protection-related proteins (**A**) filaggrin, (**B**) involucrin, (**C**) repetin, and (**D**) loricrin were measured by immunoblotting in HaCaT cells with or without pre-treatment with 1 μM of **8a C_60_(Glc)_12_**, **8b C_60_(Gal)_12_**, **8c C_60_(Man)_12_**, or non-soluble fullerene, **C_60_-H_2_O** for 1 h and then incubation with PM (50 μg/cm^2^) for 24 h. Glyceraldehyde 3-phosphate dehydrogenase (GAPDH) was used as a loading control. The blots were representative of three independent experiments. The data were collected from at least three individual experiments and expressed as mean ± SEM. ** *p* < 0.01 when compared to the PM exposure group.

Several proteins, such as filaggrin, involucrin, and loricrin, act as a barrier against the damage caused by PM in keratinocytes [5,36]. Therefore, we investigated whether glycofullerenes have a protective effect in PM exposed HaCaT cells. As shown in Figure 7, we observed a notable suppression effect on PM-induced filaggrin, involucrin, loricrin, and repetin loss (at 24 h) in cells pre-treated with 1 μM of **8a C_60_(Glc)_12_**, **8b C_60_(Gal)_12_**, and **8c C_60_(Man)_12_**, while pre-treatment with the non-soluble fullerene, **C_60_-H_2_O** did not induce any change. This indicates that the glycofullerenes could preserve the levels of protection-related proteins in keratinocytes under PM exposure.

## 4. Discussion

The present study was focused on the synthesis of water-soluble fullerenes, particularly that of glycofullerenes, and aimed to investigate their protective effects against PM-induced inflammation and oxidative stress. In the current study, the Standard Reference Material^®^ 1649b (SRM 1649b), which has been certified by the National Institute of Standards and Technology with organic constituents (such as polycyclic aromatic hydrocarbons and polychlorinated biphenyl), was selected to mimic the real effects of PM-induced damage which is attributable to urban dust on skin cells. According to the certificate of SRM 1649b, the PM was collected from the Washington, DC area in 1976 and 1977 and passed through a 63 μm filter (230 mesh). This indicates that SRM 1649b includes PM with various particle sizes (coarse, fine particles, and ultrafine particles). The use of SRM 1649b could prevent the recording of effects due to unknown compounds in the composition of PM and enable easy comparison between the results of other studies. Firstly, we discussed the synthetic procedure used, as well as the characterization of the newly synthesized glycofullerenes. As non-modified fullerenes **4** are non-soluble in water, we first synthesized glycofullerenes **8a C_60_(Glc)_12_**, **8b C_60_(Gal)_12_**, and **8c C_60_(Man)_12_** using an effective click chemistry strategy to obtain water-soluble glycofullerene derivatives (Scheme 1). FTIR measurements (Figure 1a) indicate the typical signals of fullerene derivative functional groups for the synthesis steps. Moreover, the DLS data (Figure 1b) provided direct and fast information of the hydrodynamic diameter ranges of the glycofullerenes, while TEM images were used to estimate the sizes and further provide information regarding the surface morphology (Figure 1d). The negative charge obtained by the zeta potential measurement (Figure 1c) of **8a C_60_(Glc)_12_**, **8b C_60_(Gal)_12_**, and **8c C_60_(Man)_12_** was attributed to the hydroxyl group (-OH) of the sugar moieties on the glycofullerenes. 

Previous studies have suggested that fullerenes may act as a potential antioxidant in biological systems [10]. Due to their intrinsic structure, fullerenes exhibit a unique chemical property, namely having many conjugated double bonds in the lowest unoccupied molecular orbital (LUMO). This enables easy acceptance of an electron, therefore increasing the likelihood of it attacking and neutralizing radical species [10]. The non-water-soluble fullerene (also known as an aqueous dispersion of fullerene), which was termed as **C_60_-H_2_O** is the present study, has been reported to be a powerful antioxidant with no lethal, acute or subacute effects on liver cells or animals [37,38]. Its anti-oxidant effects may occur via inactivation of hydroxyl radicals which attach to the double bonds or acquire a positive charge, as well as absorption of protons in the mitochondria [39]. However, studies have also shown that it exerts toxic effects in living systems by inducing oxidative stress, such as anti-bacterial or anti-viral effects [10]. The ROS generation induced by fullerene may be associated with light exposure and oxygen concentration [40]. Generally, fullerenes dispersed in water with no organic solvent are safe in mammals [10,38]. In the current study, we selected a dose of **C_60_-H_2_O** (with no organic solvent) which would not cause significant effects in our test platform in order to identify the potential effects of glycofullerenes and non-water-soluble fullerene. All cellular biomacromolecules, including lipids, sugars, proteins, and DNA, can react with ROS. However, the generated secondary ROS products can be even more dangerous to the cell than the initially formed ROS. In normal condition, cells can defend themselves against ROS damage by using either enzymes or small antioxidant molecules. These enzymes can be catalase, superoxide dismutase, lactoperoxidase, peroxiredoxin, and glutathione peroxidase, while small antioxidant molecules can be α-tocopherol (vitamin E), vitamin C, and glutathione. ROS play a role as a double-edged sword, as they maintain redox homeostasis in physiological conditions, while also being involved in various pathological processes in the cell [41,42,43]. Under physiological conditions, ROS are involved in phagocytosis, activation of downstream cell signaling, and maintaining homeostasis. In this case, ROS levels are regulated by endogenous cells scavenging systems which neutralize and eliminate excess ROS [44,45]. However, under unbalanced, oxidative stress conditions, cellular lipids, proteins, and DNA can be oxidized by higher concentrations of ROS. Subsequently, several diseases and phenomena can be promoted or induced by excess ROS, such as tissues inflammation, organ aging, neurodegeneration, and cancer [45,46,47,48,49]. PM can cause aberrant ROS accumulation [5,33]. Here, we used 1 μM of glycofullerenes **8a C_60_(Glc)_12_**, **8b C_60_(Gal)_12_**, and **8c C_60_(Man)_12_**, respectively, and non-water-soluble **C_60_-H_2_O** as a comparison to study their protective effects against PM-induced inflammation and oxidative stress. The results of the cell apoptosis assays were first to reveal that **8a C_60_(Glc)_12_**, **8b C_60_(Gal)_12_**, **8c C_60_(Man)_12_**, and non-water-soluble **C_60_-H_2_O** did not significantly induce apoptosis of HaCaT cells at the concentration used in this study, while PM (50 μg/cm^2^) treatment did. Notably, our results showed that pre-treatment with all of the glycofullerenes could suppress PM-triggered early-apoptosis, while **C_60_-H_2_O** pre-treatment had no effect. 

The major advantage of water-soluble fullerenes for use as an antioxidant in the medical field is their potential ability to localize within the cell to the mitochondria [50] and other cellular compartments, where the production of free radicals is increased. Here, cellular ROS production was measured via H2DCFDA and CellROX staining (Figure 3). All cellular ROS assays performed indicated that PM exposure significantly raised ROS levels in the cell, while glycofullerenes pre-treatment could reduce PM-induced ROS production. However, this suppression effect could not be observed in the group treated with non-soluble fullerene **C_60_-H_2_O**. Furthermore, we used MitoSOX staining (Figure 4) to detect the levels of ROS generated by the mitochondria. As expected, glycofullerenes were found to effectively decrease PM-induced ROS production in the mitochondria, while **C_60_-H_2_O** did not. 

The MAPK superfamily consists of three main protein kinase families: ERKs, c-JNKs, and p38. Each was shown to play major roles in the regulation of the intracellular metabolism and gene expression. Moreover, they play a critical role in regulating various cellular processes, such as gene expression, mitosis, differentiation, apoptosis, cellular responses to external stresses, and disease [51]. These three main kinases of the MAPK family may be regulated by different signaling cascades and play different roles. For example, phospho-ERK1/2 plays an essential role in the downstream phosphorylation of p38 in HaCaT cells. In most mammalian tissues, ERK2 was shown to have a higher expression level than ERK1, which may be due to the strength of its proximal promoter [52]. Studies have also suggested that ERK1 and 2 may play different roles in various cells and experimental conditions [52]. Furthermore, our previous study suggested that PM-stimulated phosphorylation of ERK1/2, JNK1/2, and p38 was mediated by ROS in HaCaT cells [5,29]. This indicates that the activation of the MAPK pathways is associated with PM-induced increase in ROS, as well as inflammatory processes in skin cells. In this study, the PM-activated MAPK pathways could be suppressed by pre-treatment with glycofullerenes (Figure 5). However, in the current stage, our results could not distinguish between the effects of glycofullerene treatment on the individual regulatory roles of ERK1 and 2 in PM-exposed HaCaT cells, due to the similarity between the regulation patterns of ERK1 and 2 after PM and glycofullerenes treatment. Additionally, JNK-1 and -2 were also shown to play different roles in various conditions [53]. Under normal conditions, in several cells and animal models (such as the proliferation of keratinocytes in the mouse skin), JNK1 acts as a positive regulator, while JNK2 exhibits a negative or downregulatory function [54]. JNK-2 has been also reported to be more important than JNK-1 in some skin diseases, such as human squamous cell carcinoma [55]. However, JNK-1 was found to be more affected than JNK-2 in our PM-exposure cell model. We hypothesized that the increase in JNK-1 levels following PM exposure may lead to resistance to PM-induced damage. However, PM is a mixture of components and may induce complicated effects on signal transduction. Hence, our findings could not identify the individual roles of JNK-1 and 2 in PM-induced inflammation in skin cells and, therefore, require further investigation. The Akt pathway is important for skin aging [56] and has been suggested to promote tissue regeneration and wound healing [57,58]. Loss of Akt leads to a thinner epidermal layer and delayed development of the hair follicle [59]. These findings indicate that the MAPK and Akt pathways are involved in the mechanisms which protect skin cells from damage induced by environmental toxins. As such, maintenance of the MAPK and Akt pathways within physiological conditions could help maintain the health of skin cells. In the current study, our results suggested that glycofullerenes could dramatically suppress PM-induced phosphorylation of MAPKs and Akt proteins, while **C_60_-H_2_O** had no effect. These results imply that the water-solubility of glycofullerenes may play a critical role in their bioactivity and signal transduction for skin cells. 

Previously, we found that the MAPK signaling pathways were involved in the PM-induced ROS-mediated COX-2/PGE2 and MMP-9 inflammation pathway [5,29]. Previous studies have reported that HO-1 may be induced by oxidative or nitrosative stress [60] and play antioxidant and anti-inflammatory roles in various pathological conditions [61,62,63]. In addition, it is well known that ICAM-1 is highly expressed during lymphoid infiltration of epidermal keratinocytes and plays an important role in inflammatory-related mechanisms, such as lymphocyte transendothelial migration [64,65,66]. cPLA2 is an enzyme which hydrolyzes phospholipids to produce arachidonic acid or lysophospholipids and is also a type of inflammatory factor. Notably, cPLA2 inhibitors may be potential therapeutic agents for inflammatory skin diseases [67,68]. Our results revealed that glycofullerenes could inhibit the expression of COX-2, HO-1, MMP-9, ICAM-1, and cPLA2, as well as the production of PGE2, induced by PM treatment, while **C_60_-H_2_O** did not (Figure 6). Furthermore, PM exposure can induce an increase in COX-2 expression and decrease in filaggrin expression [5]. Filaggrin, repetin, involucrin, and loricrin act as barrier/protective proteins in keratinocytes to help the skin defend against the external environment (pathogens and allergens entry or chemical damage) and reduce water loss [69]. Moreover, filaggrin levels have been shown to be linked with the activation of inflammatory pathways [70]. Therefore, maintaining the expression level of filaggrin, repetin, involucrin, and loricrin protects the keratinocytes against the damage caused by PM [5,36]. Here, we further revealed that our glycofullerenes could maintain the level of protection-related proteins in keratinocytes under PM exposure (Figure 7). This result proves that glycofullerenes are promising potential protective agents against PM-induced skin damage. 

## 5. Conclusions

**Figure 8:**
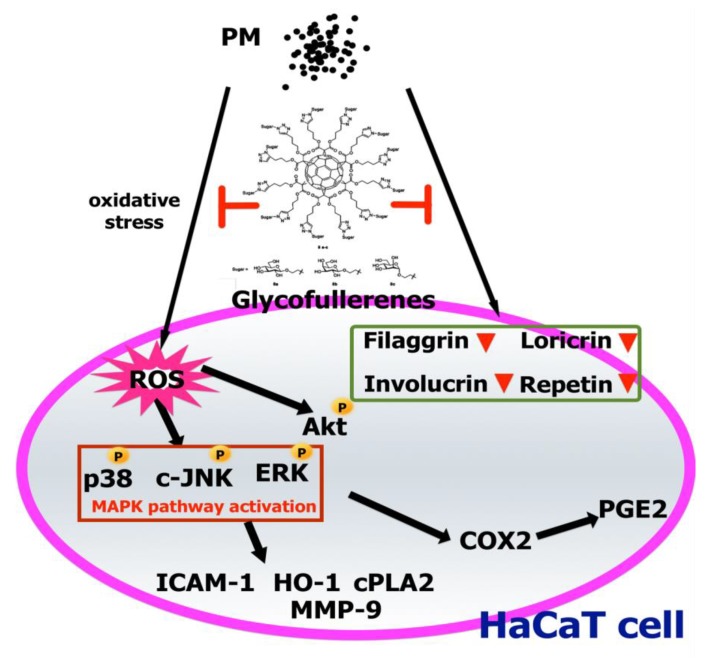
A model for the molecular mechanisms underlying the suppressive effects of glycofullerenes on particulate matter (PM)-triggered oxidation, inflammation, and loss of barrier proteins in HaCaT cells. PM increased ROS generation and activated the MAPK and Akt pathways, followed by upregulation of the expressions of inflammatory proteins (ICAM-1, COX-2, HO-1, and PGE2, etc.). Additionally, the levels of barrier proteins were decreased by PM exposure. Finally, cell apoptosis occurred. Pre-treatment with glycofullerenes could inhibit the PM-induced adverse effects via mechanisms such as ROS scavenging, anti-inflammation, and maintenance of the expressions of barrier proteins in HaCaT cells.

The three glycofullerenes designed in this study, **8a C_60_(Glc)_12_**, **8b C_60_(Gal)_12_**, and **8c C_60_(Man)_12_**, were shown for the first time to have good protective effects against PM-induced oxidative stress, inflammation, and impairment of barrier proteins in HaCaT keratinocytes (Figure 8). In contrast, the poor water-solubility of **C_60_-H_2_O** may be the cause of its lack of antioxidant and anti-inflammation activity, and barrier proteins preservation effects. The glycofullerenes may have great potential applications as cosmeceutical products to protect the skin from PM-induced inflammation and oxidative stress damage or as medicine to treat ROS-caused skin diseases in the future.

## Supplementary Materials

The following are available online at https://www.mdpi.com/2218-273X/10/4/514/s1, Figure S1: NMR spectrum of compound **3**, Figure S2: NMR spectrum of compound **5**, Figure S3: IR spectra of compound **5**, Figure S4: UV spectra of compound **5** in DMSO, Figure S5: NMR spectrum of compound **6**, Figure S6: IR spectra of compound **6**, Figure S7: UV spectra of compound **6** in DMSO, Figure S8: Synthetic route of glucoside **7a**, galactoside **7b**, and mannoside **7c**, Figure S9: NMR spectrum of compound **8a**, Figure S10: IR spectra of compound **8a**, Figure S11: UV spectra of compound **8a** in DMSO, Figure S12: Mass spectra of compound **8a**, Figure S13: NMR spectrum of compound **8b**, Figure S14: IR spectra of compound **8b**, Figure S15: UV spectra of compound **8b** in DMSO, Figure S16: Mass spectra of compound **8b**, Figure S17: NMR spectrum of compound **8c**, Figure S18: IR spectra of compound **8c**, Figure S19: UV spectra of compound **8c** in DMSO, Figure S20: Mass spectra of compound **8c**.

## Abbreviations

AP-1 COX-2	Activator protein 1 Cyclooxygenase
cPLA2 DLS	Cytosolic phospholipases A2 Dynamic light scattering
ERK	Extracellular regulated protein kinase
FITC	Fluorescein isothiocyanate
GAPDH H2DCFDA	Glyceraldehyde 3-phosphate dehydrogenase 2′,7′-dichlorodihydrofluorescein diacetate
HO-1	Heme oxygenase-1
ICAM-1 IR	Intercellular adhesion molecular-1 Infrared spectroscopy
JNK LUMO	c-Jun N-terminal kinase Lowest unoccupied molecular orbital
MAPK	Mitogen-activated protein kinase
MMP-9 NF-κB NMR	Metalloproteinase-9 nuclear factor kappa-light-chain-enhancer of activated B cells Nuclear magnetic resonance spectroscopy
PM	Particulate matter
PGE2	Prostaglandin E2
ROS TEM TLC UV-Vis	Reactive oxygen species Transmission electron microscopy Thin-layer chromatography Ultraviolet–visible spectroscopy
