# Supplementary material for: Glycofullerenes Inhibit Particulate Matter Induced Inflammation and Loss of Barrier Proteins in HaCaT Human Keratinocytes
Source: Biomolecules. 2020 Mar 28;10(4):514. doi: 10.3390/biom10040514 (PMC7225947; doi:10.3390/biom10040514)
Supplement: Supplementary file 1 [file biomolecules-10-00514-s001.pdf]

Supplementary Data

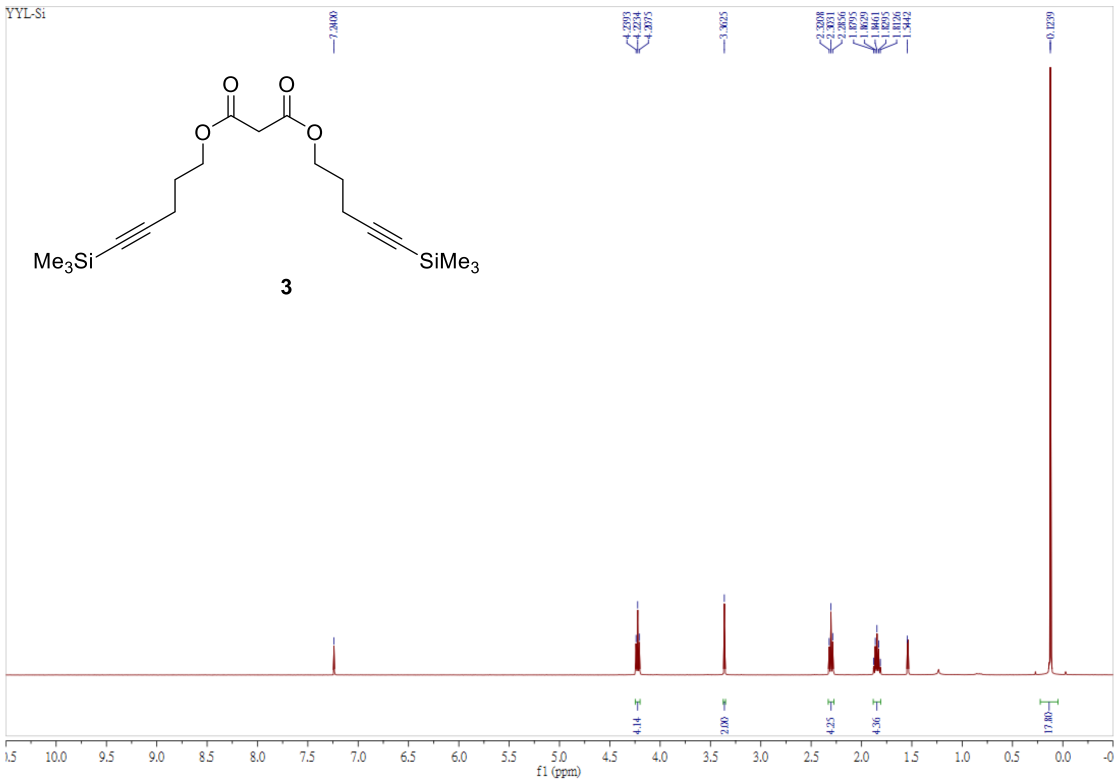

Figure S1. NMR spectrum of compound 3

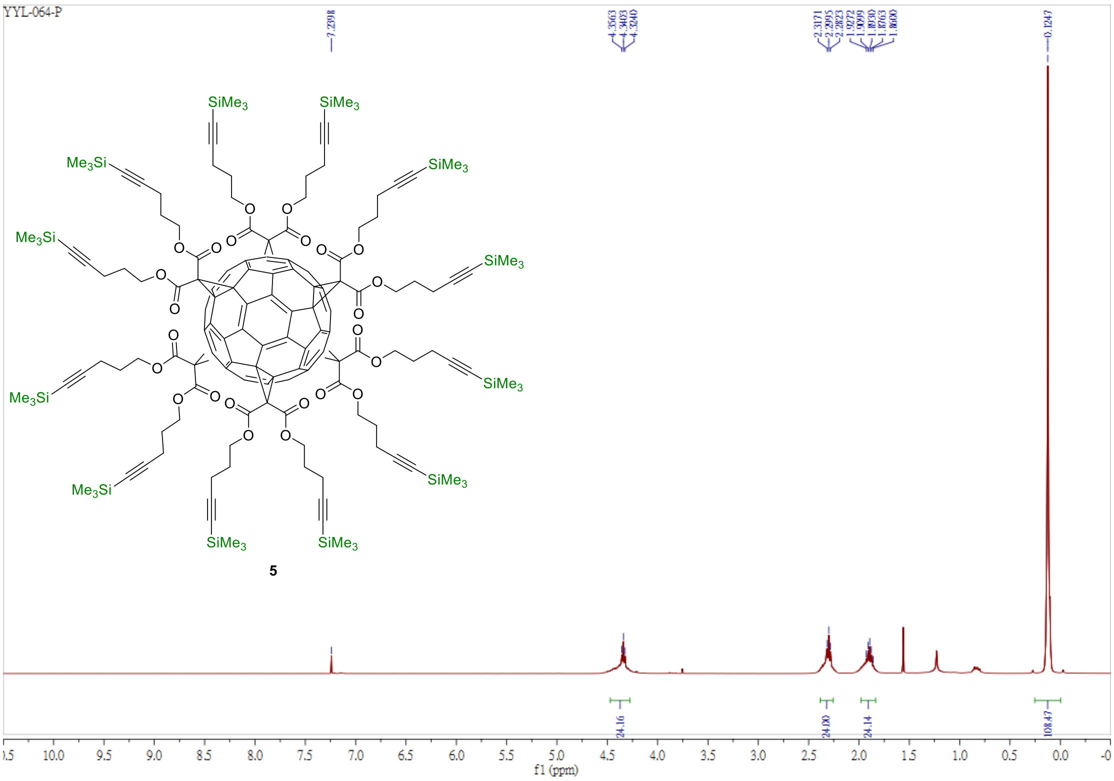

Figure S2. NMR spectrum of compound 5

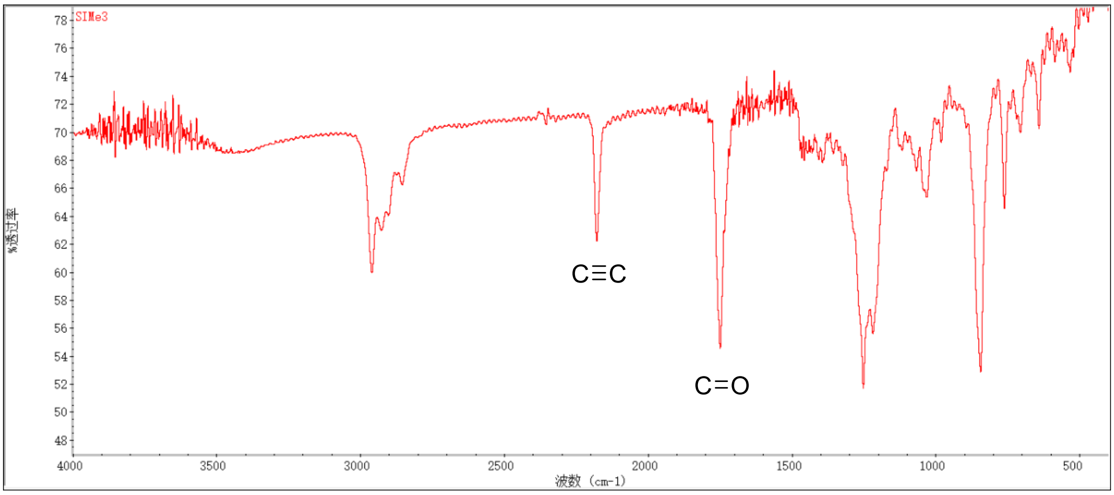

Figure S3. IR spectra of compound 5.

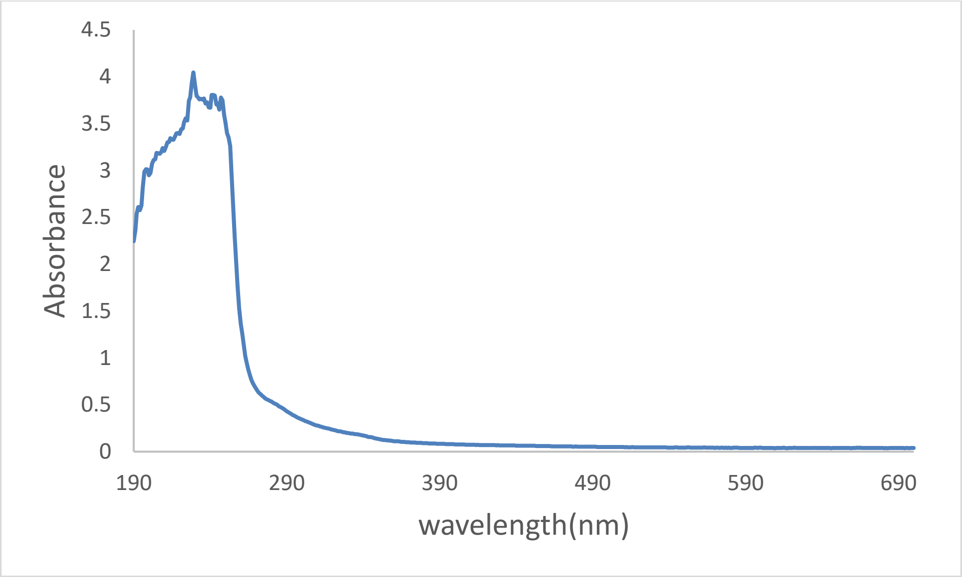

Figure S4. UV spectra of compound 5 in DMSO.

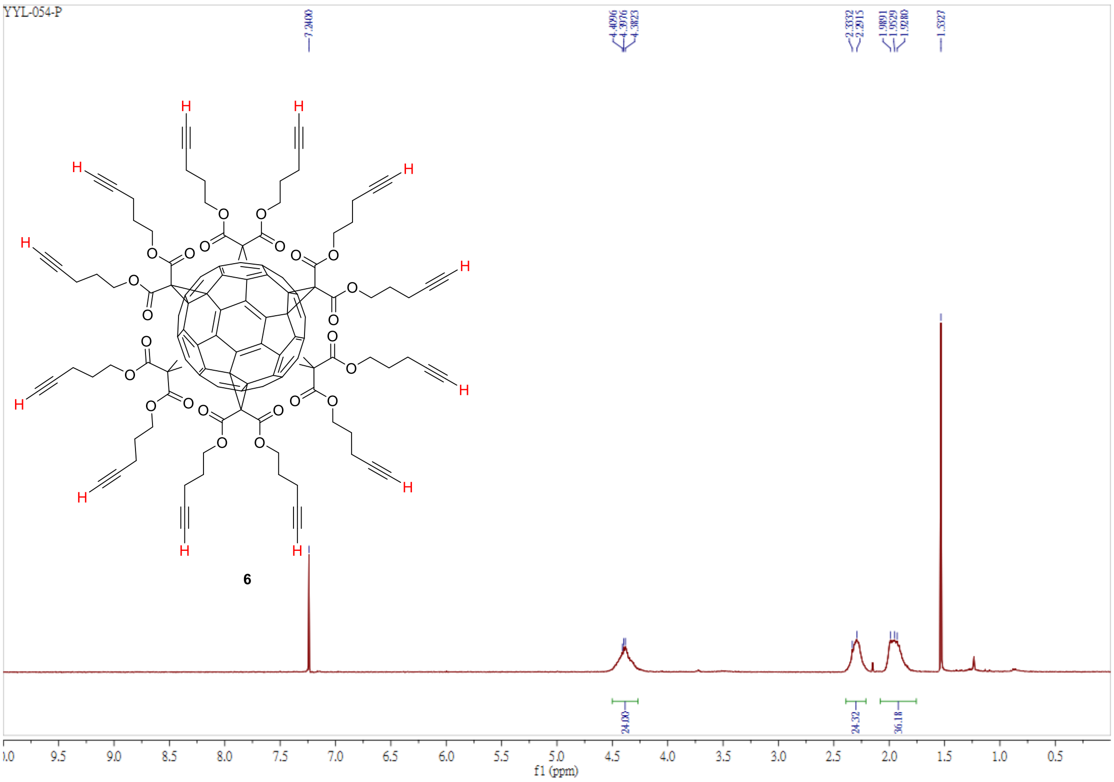

Figure S5. NMR spectrum of compound 6

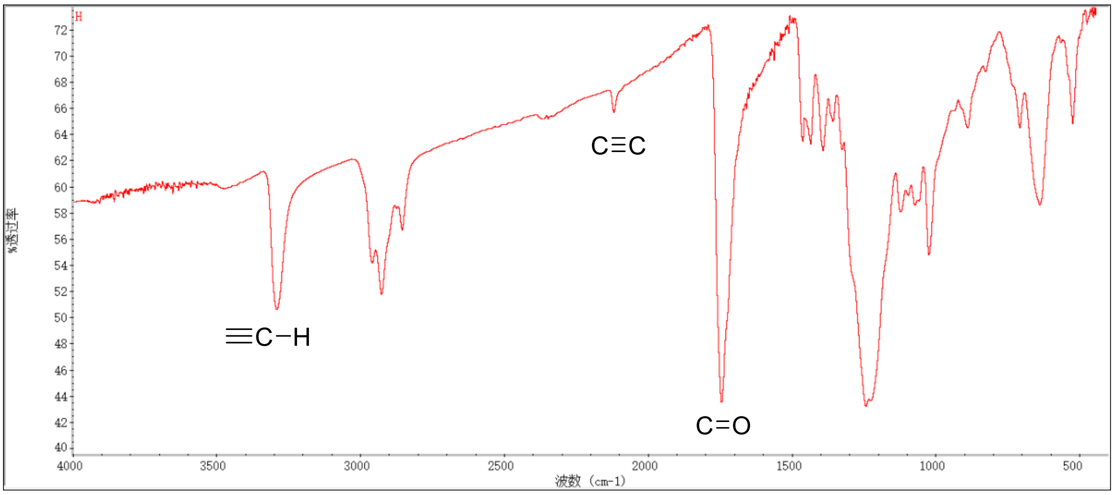

Figure S6. IR spectra of compound 6.

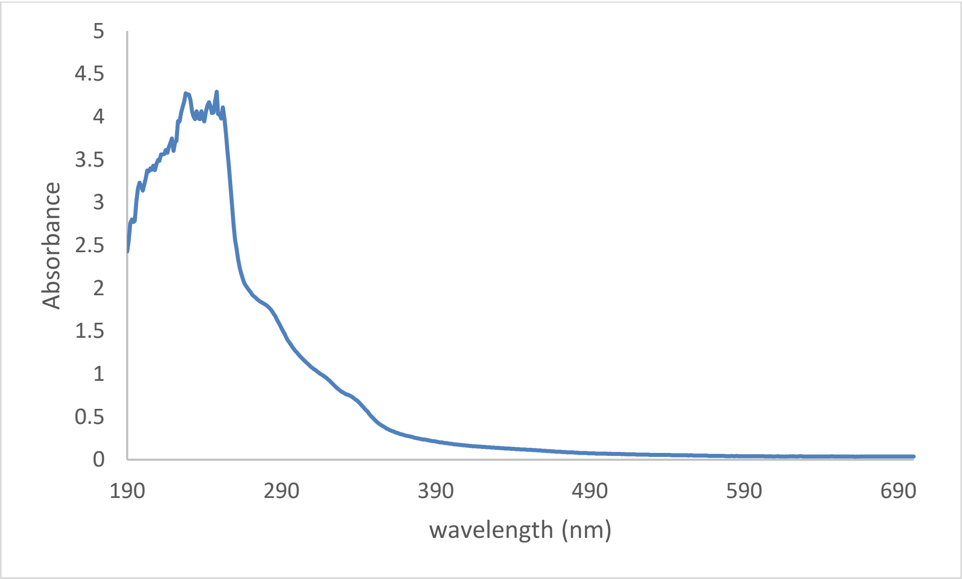

Figure S7. UV spectra of compound 6 in DMSO.

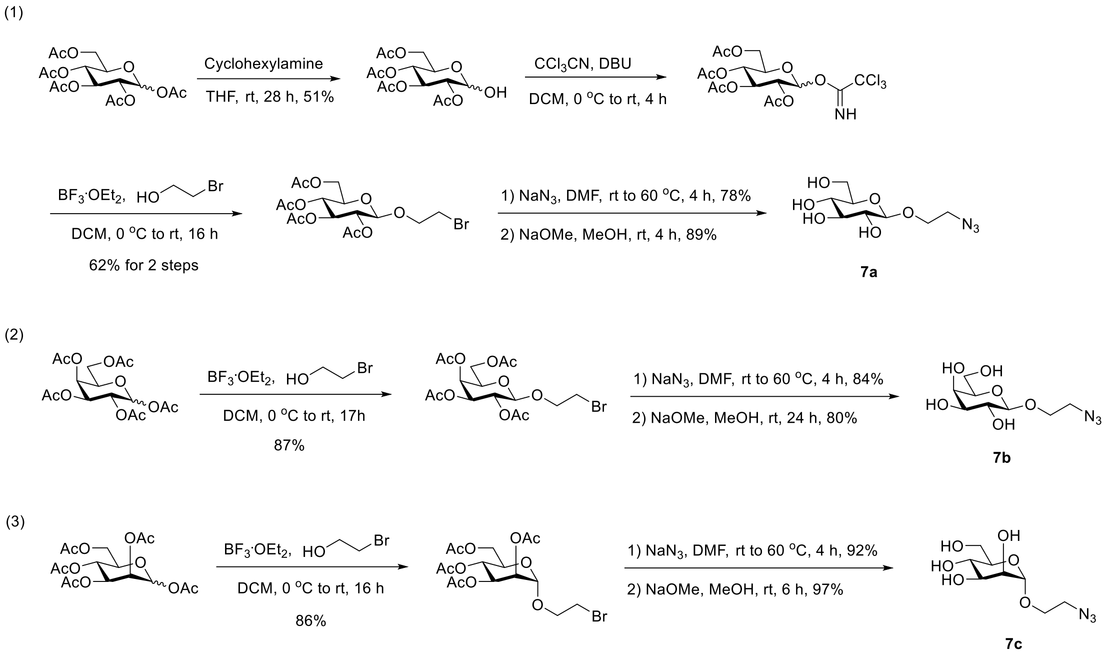

Figure S8. Synthetic route of glucoside 7a, galactoside 7b and mannoside 7c.

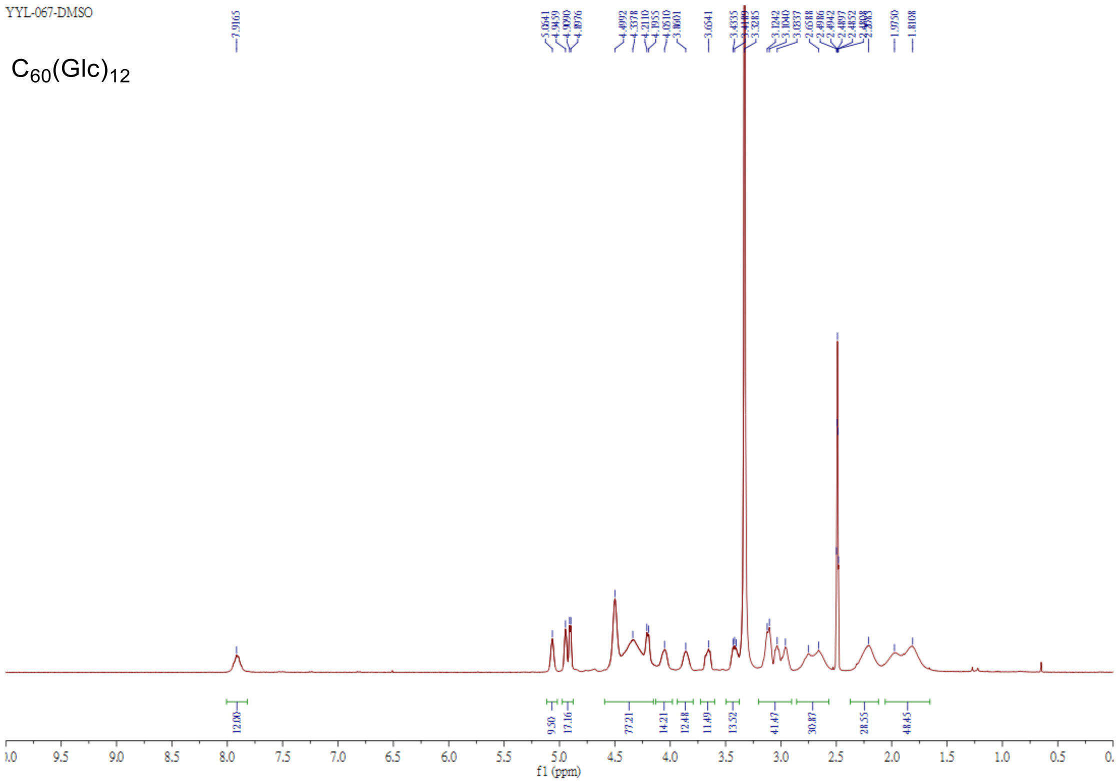

Figure S9. NMR of compound 8a

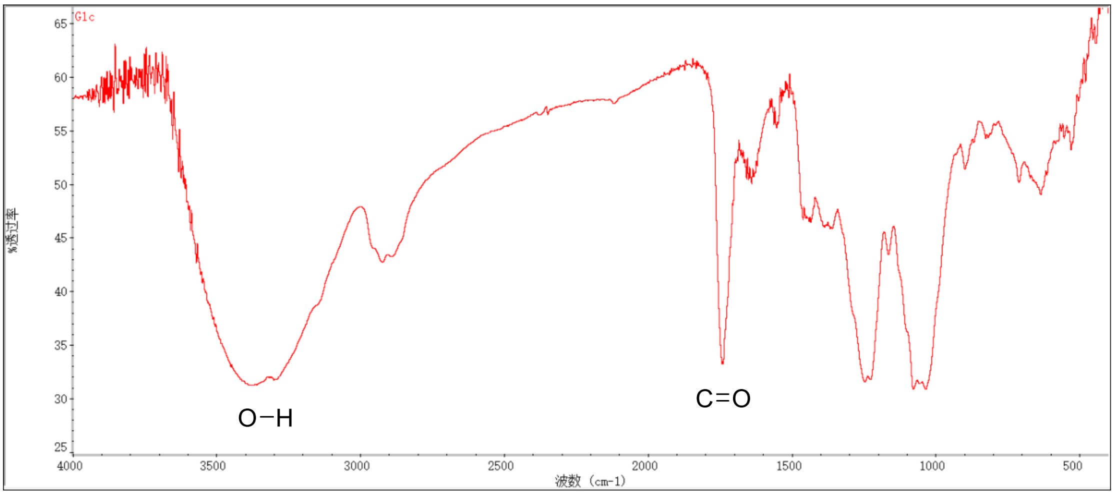

Figure S10. IR spectra of compound 8a.

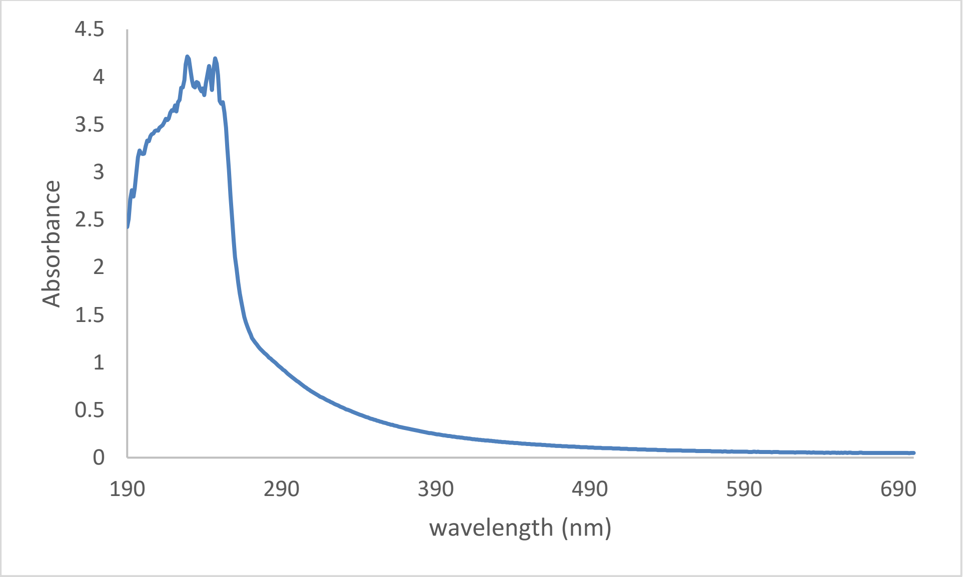

Figure S11. UV spectra of compound 8a in DMSO.

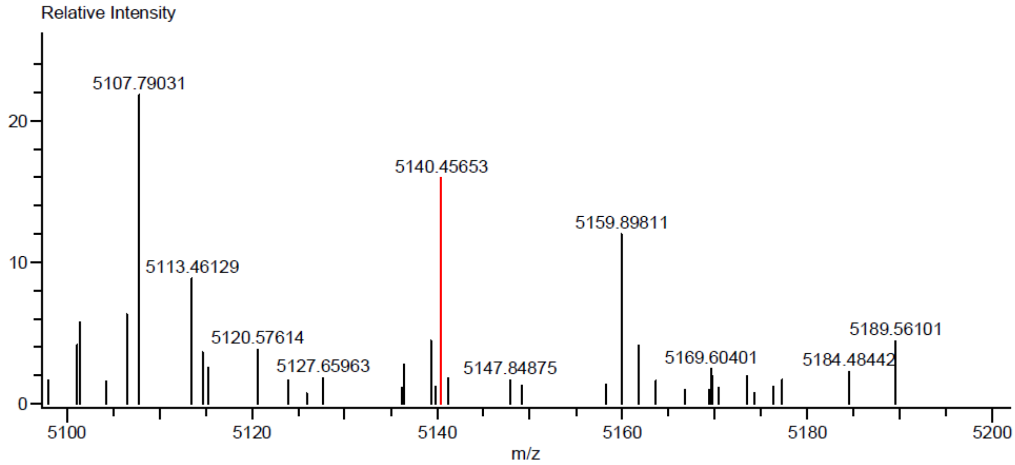

Figure S12. Mass spectra of compound 8a.

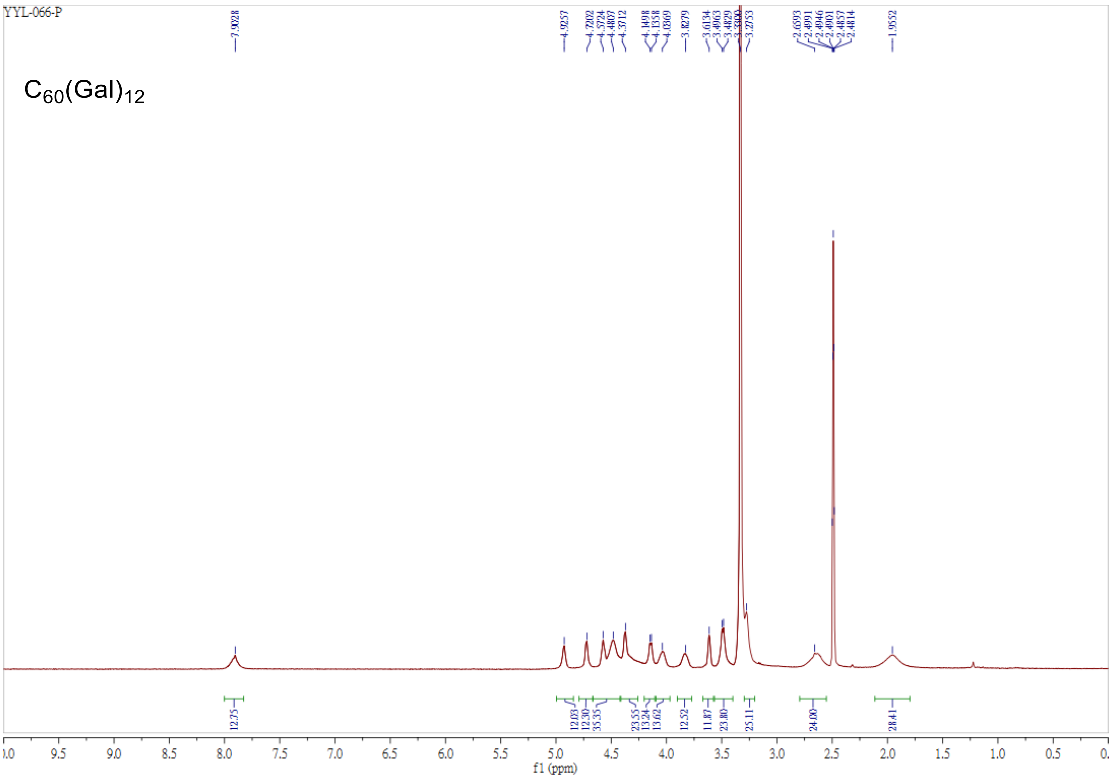

Figure S13. NMR of compound 8b

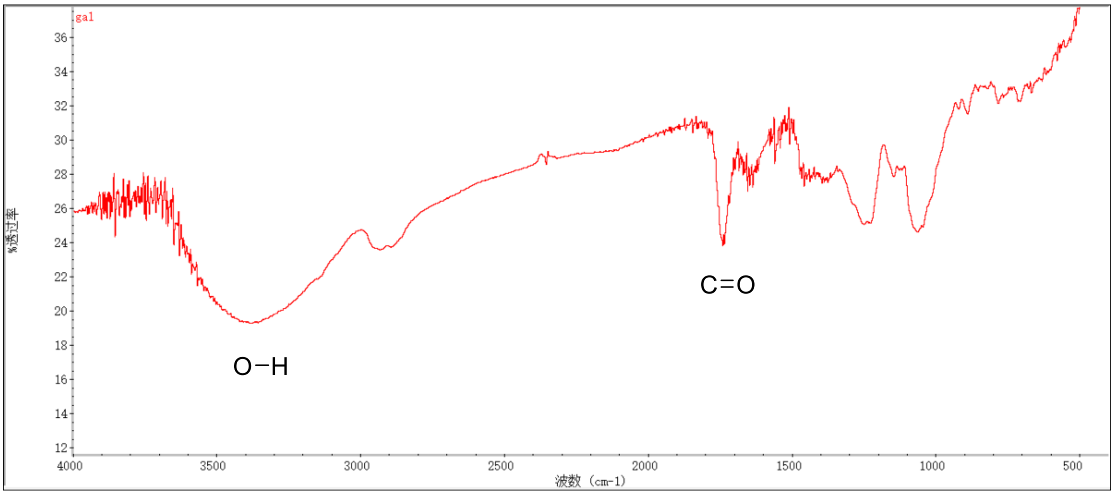

Figure S14. IR spectra of compound 8b.

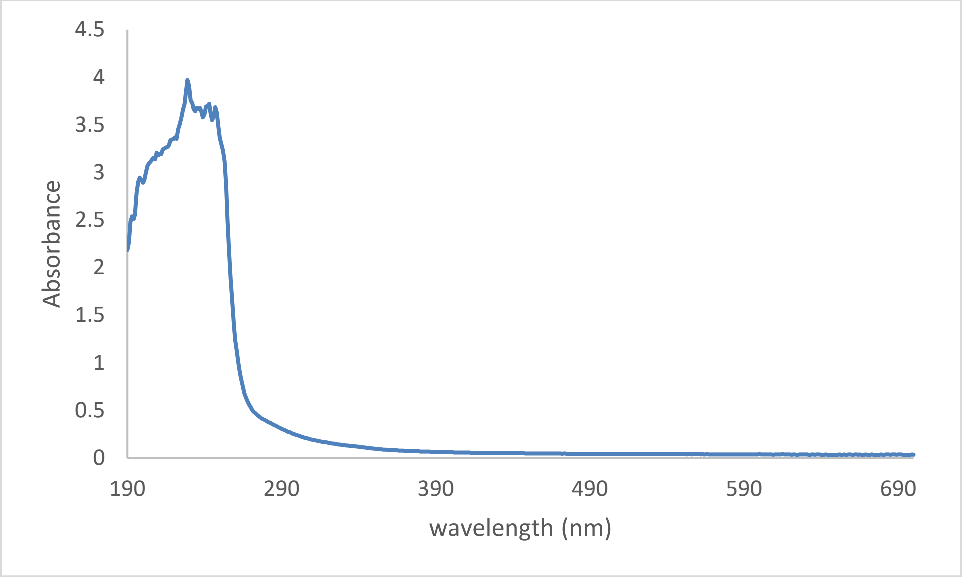

Figure S15. UV spectra of compound 8b in DMSO.

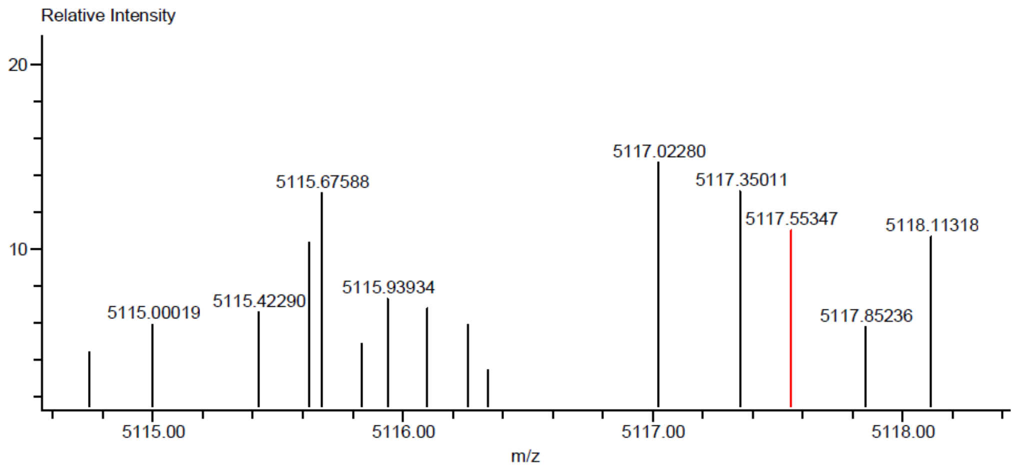

Figure S16. Mass spectra of compound 8b.

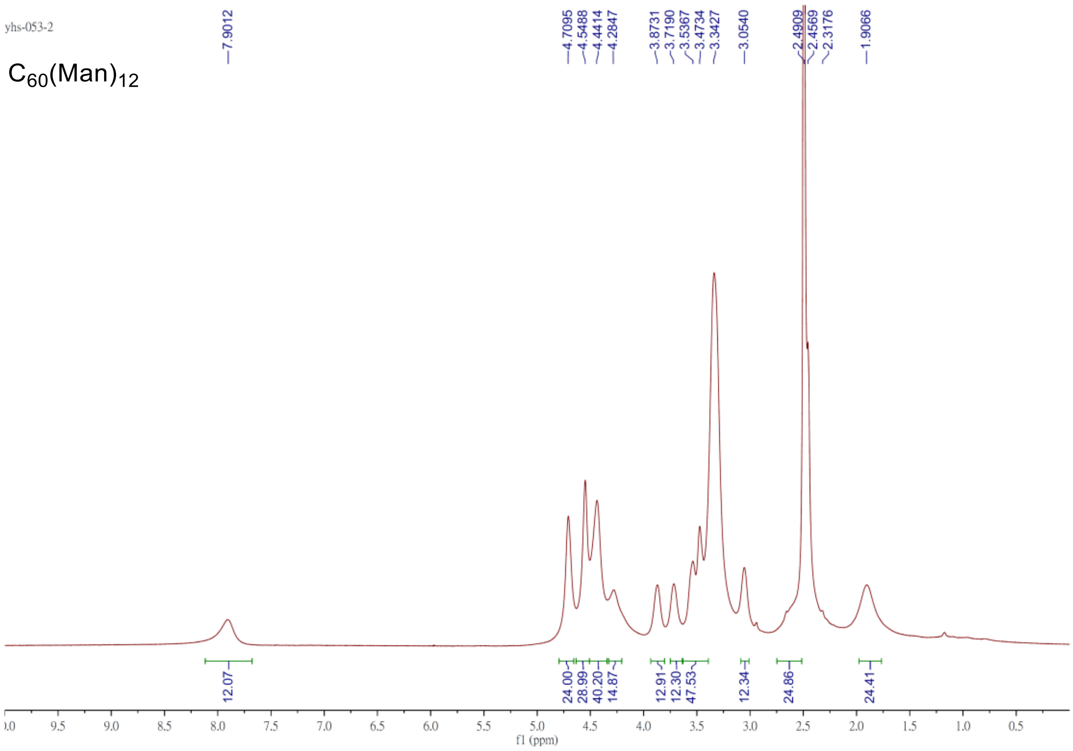

Figure S17. NMR of compound 8c

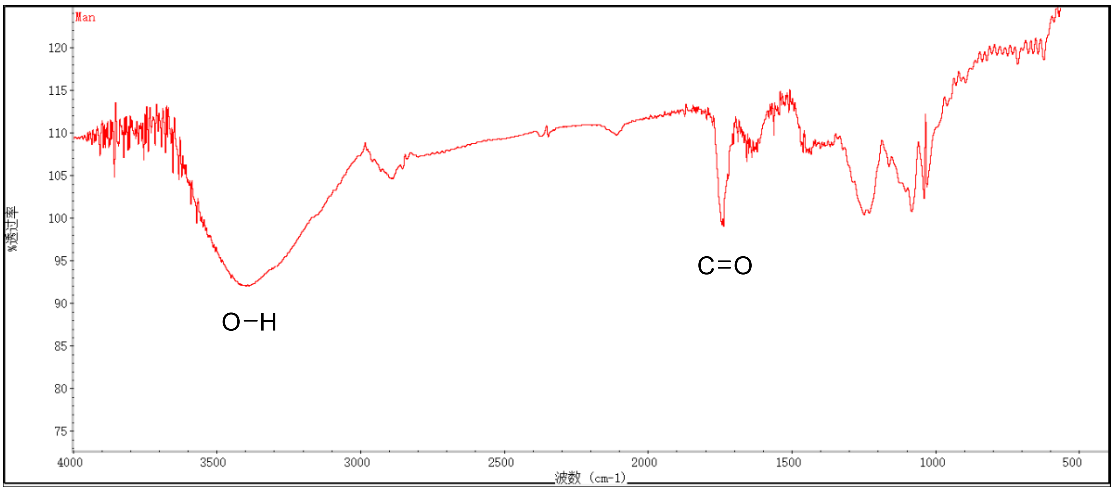

Figure S18. IR spectra of compound 8c.

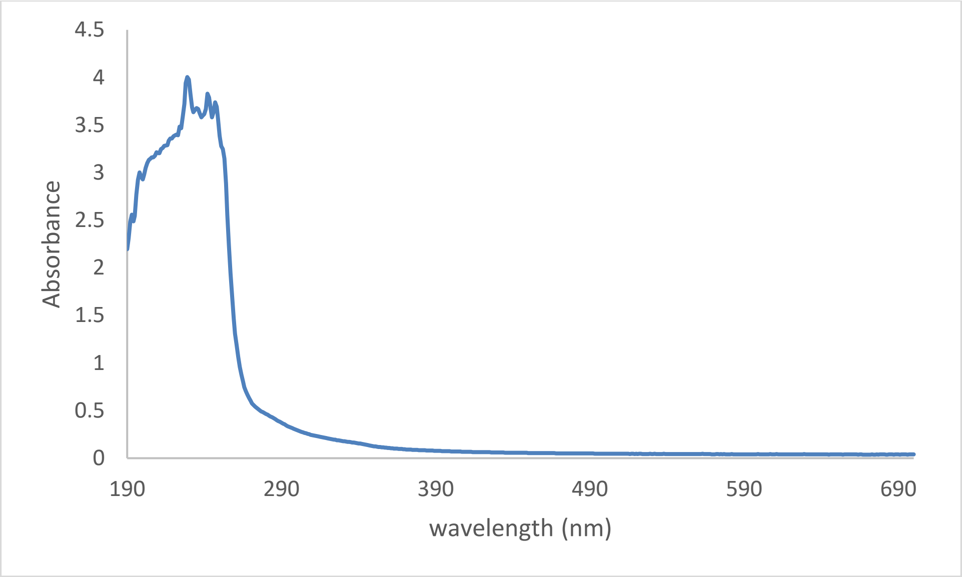

Figure S19. UV spectra of compound 8c in DMSO.

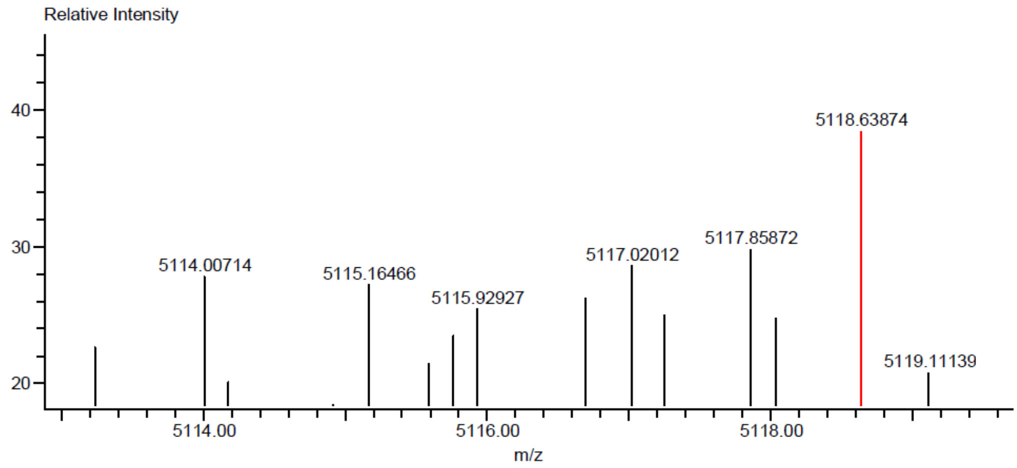

Figure S20. Mass spectra of compound 8c.
